# Cap‐Drop: A Pre‐Programmed, Self‐Powered Capillary Microfluidic System for Passive Droplet Generation and 3D Cell Culture Modeling

**DOI:** 10.1002/smll.202411997

**Published:** 2025-05-22

**Authors:** Pezhman Jalali, Bahareh Zarin, Azam Zare, Sorosh Abdollahi, Mohsen Hassani, Maryam Vatani, Mohammadreza Farrokhnia, Razieh Salahandish, Hossein Hejazi, Amir Sanati‐Nezhad

**Affiliations:** ^1^ BioMEMS and Bioinspired Microfluidic Laboratory Department of Biomedical Engineering University of Calgary Calgary Alberta T2N 1N4 Canada; ^2^ Department of Chemical and Petroleum Engineering University of Calgary Calgary Alberta T2N 1N4 Canada; ^3^ Department of Mechanical and Manufacturing Engineering University of Calgary Calgary Alberta T2N 1N4 Canada

**Keywords:** 3D cell culture, capillary microfluidics, droplet arrays, high‐throughput screening, microwell arrays

## Abstract

3D cell culture models and precision diagnostics have advanced significantly through microfluidic systems, yet their broad implementation remains limited by challenges in scalability, integration, and portability. Effective 3D cell culture models require systems that maintain sample integrity, minimize evaporation, and avoid crosstalk while handling various biofluids. However, current platforms often depend on active pumping, bulky components, and complex controls, which hinder portability, usability, and affordability. To address these challenges, the Capillary Droplet microfluidic (Cap‐Drop) is presented, a novel capillary‐driven platform that generates and immobilizes droplets with precision, eliminating the need for external pumps or intricate setups. Unlike conventional system, where moving droplets complicate tracking and identification, Cap‐Drop ensures fixed droplet positioning, allowing seamless tracking and analysis. By integrating hydrophilic and hydrophobic materials with several innovative capillary elements —including passive vents (PV), pressure reducer (PR), stop valves (SV), delay channels, and bubble trap (BT)—Cap‐Drop enables robust droplet formation (40 to 500 nL) for biofluids of varying properties. The pre‐programmed design of PV in corporation with other capillary elements autonomously seals microwells (MWs), ensuring consistent sample digitization and supress risk of evaporation. Cap‐Drop is optimized and offers a transformative platform for microfluidic technologies in mechanistic cellular studies, preclinical drug screening, and clinical diagnostics.

## Graphical abstract

In this work, a novel capillary droplet microfluidic system(Cap‐Drop) is introduced that overcomes major limitations of diagnostic platforms by offering a portable and pump‐free solution for precise sample digitization with 3D cell culture application. The system's multilayer structure facilitates the integration of materials with different capillary properties, enabling strong capillary driving forces and functional capillary elements without additional surface treatment.

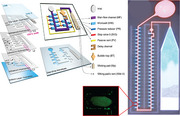

## 1 Introduction

Droplet microfluidic systems have revolutionized applications in 3D cell culture models for mechanistic studies and drug screening,^[^ ^1^, ^2^ ^]^ proteomics and protein structural analysis,^[^ ^3^ ^]^ single‐cell genomic profiling,^[^ ^4^, ^5^, ^6^, ^7^ ^]^ colloidal cross‐linked microgel assemblies,^[^ ^8^ ^]^ and precise quantification of biomolecules, including nucleic acids, hormones, metabolites, and functional and structural proteins.^[^ ^9^ ^]^ By enabling the production and manipulation of nano‐volume droplets, these systems facilitate rapid heat and mass transfer through their high surface‐to‐volume ratio. This enhances assay sensitivity, reduces sample consumption, and improves efficiency, making droplet microfluidics a cornerstone of modern precision diagnostics and scalable 3D culture models for drug screening.

A well‐established method for throughput, monodisperse droplet generation involves dispersing an initial phase including sample solution into an immiscible phase at predefined flow ratios, with careful control over droplets size and generation mode.^[^ ^10^ ^]^ Various mechanisms have been emerged based on this method over the past two decades, such as step emulsification,^[^ ^11^, ^12^, ^13^, ^14^ ^]^ cross flow,^[^ ^15^ ^]^ and flow focusing,^[^ ^16^, ^17^ ^]^ each extensively discussed in recent reviews.^[^ ^18^ ^]^ Despite their potential, the reliance on active pumps, e.g., syringe,^[^ ^11^, ^15^, ^16^, ^17^, ^19^ ^]^ or centrifugal pumps,^[^ ^12^, ^13^, ^14^ ^]^ to create positive pressure, combined with the challenges of monitoring droplets in motion and the need for complex fabrication processes, poses barriers to their ease of use for users in diverse fields and hinder their implementation in advanced point‐of‐care diagnostics and wearable applications. Passive flow techniques have been investigated in two‐phase flow systems to eliminate the need for active pumps, leading to the development of finger‐actuated systems^[^ ^20^ ^]^ and handheld pumps.^[^ ^21^ ^]^ However, these methods still require further refinement to effectively control the two‐phase flow ratios at junction points.

Another approach for droplet generation, known as droplet array or sample digitization, has been introduced in different studies.^[^ ^22^, ^23^, ^24^, ^25^, ^26^, ^27^, ^28^, ^29^, ^30^, ^31^, ^32^, ^33^, ^34^, ^35^, ^36^, ^37^ ^]^ This method eliminates the need for a two‐phase collision step and facilitating the trapping and fixation of droplets in predetermined locations, enabling easy and precise monitoring.^[^ ^22^ ^]^ One well‐known example is the use of droplet arrays that can be directly patterned via inkjet printing with high precision.^[^ ^23^, ^24^ ^]^ However, the use of such set‐ups is limited due to the narrow range of suitable printing materials with appropriate rheological properties, such as viscosity and interfacial tension, to prevent clogging in the printing nozzles. Electrowetting‐based approaches, while enabling throughput droplet generation using electrical voltage and frequency, still rely on bulky active actuators and non‐scalable fabrication processes.^[^ ^25^ ^]^ Additionally, these systems are often made from non‐transparent materials, which hinder live monitoring of biological samples and species using optical microscopes, leaving sample extraction as the user's only option for further analysis.^[^ ^26^ ^]^ Forming sessile droplet arrays by creating hydrophilic patterns on hydrophobic substrates provides a straightforward and quick method for droplet generation by adjusting the wettability of the hydrophilic regions.^[^ ^27^ ^]^ Still, this technique faces challenges like rapid droplet evaporation and fluid sticking to the substrate, which can affect performance.

To eliminate the reliance of droplet array method on active pumps, actuators and 3D printers, vacuum‐battery system has been developed using degassing techniques.^[^ ^28^, ^29^, ^30^, ^31^, ^32^, ^33^, ^34^ ^]^ While they offer multiple advantages,^[^ ^38^ ^]^ such as chip transparency, suitability for droplet live monitoring, and low‐cost passive pumping, vacuum‐based systems deal with the risk of vacuum loss over time and not scalable fabrication process due to their reliance on polydimethylsiloxane (PDMS), a gas‐permeable material.^[^ ^39^ ^]^ Capillary pump techniques as second solution for generating passive driving force has been used for droplet array generation in both open and close microfluidic systems. Although using capillary pump is appealing for certain applications in open microfluidic, such system face challenges such as high evaporation rates and lower droplet throughput.^[^ ^40^ ^]^ To address these issues in closed microfluidic systems, capillary pumps applied to the sample solution using non‐gas permeable materials eliminate possible evaporation.^[^ ^41^ ^]^ However, this approach has limited versatility in applications due to its reliance on geometry and the properties of the sample solution.

We have identified our Cap‐Drop as a new capillary‐based droplet microfluidic platform that may enable reliable, self‐powered generation and positioning of droplets in a throughput, simple, user‐friendly design, eliminating the need for any external actuators. Unlike conventional droplet microfluidic systems, which face challenges in tracking and identifying moving droplets, Cap‐Drop provides fixed droplet positioning, enabling unambiguous tracking and effortless analysis. This versatile system demonstrates broad applicability, from throughput 3D cell culture models for advanced drug testing to the digitization of various biofluids with different mechanical properties. It accommodates a wide range of biofluids, including sweat, blood, and saliva, showcasing its adaptability for diverse biomedical and diagnostic applications. Droplet arrays are generated upon introducing the sample solution to the Cap‐Drop inlet, benefiting from a fast‐loading time compared to previous vacuum and capillary based system.^[^ ^28^, ^41^ ^]^

The Cap‐Drop ensures reliable, bubble‐free droplet generation with no evaporation or contamination, thanks to strategically integrated capillary elements including passive vents (PV), stop valves (SV), pressure reducers (PR), delay channels, and bubble trap(BT). The flexible, transparent, and biocompatible design offers a robust, scalable solution for droplet array generation using low‐cost materials. As a proof of concept, Cap‐Drop produced a throughput array of >100 droplets, each containing an intestinal cell‐laden microgel spheroid confined to its own microwell (MW). these droplets were cross‐linked in situ, and cultured on‐chip for 48 h, allowing real‐time monitoring of cell viability and functionality. Compared to commercial high‐throughput platforms for spheroid and organoid generation—such as AggreWell microwells,^[^ ^42^ ^]^ Corning ULA plates^[^ ^43^ ^]^ InSphero hanging drop systems,^[^ ^44^ ^]^ and CELLINK droplet bioprinting setups^[^ ^45^ ^]^—Cap‐Drop offers a simpler and more accessible alternative. These conventional systems often depend on robotic pipetting, external actuators, or bioprinters for droplet placement and cell handling. In contrast, Cap‐Drop uses a fully passive, self‐powered mechanism to autonomously generate, position, and isolate droplets, eliminating the need for external equipment. This enables rapid prototyping and parallel culture of 3D cell constructs in a compact, low‐cost format. Its transparency, modularity, and on‐chip compatibility with imaging and analysis makes it especially well‐suited for early‐phase drug screening and mechanistic assays.

## 2 Results and Discussion

### 2.1 Design and Working Principle of Cap‐Drop

**Figure 1:**
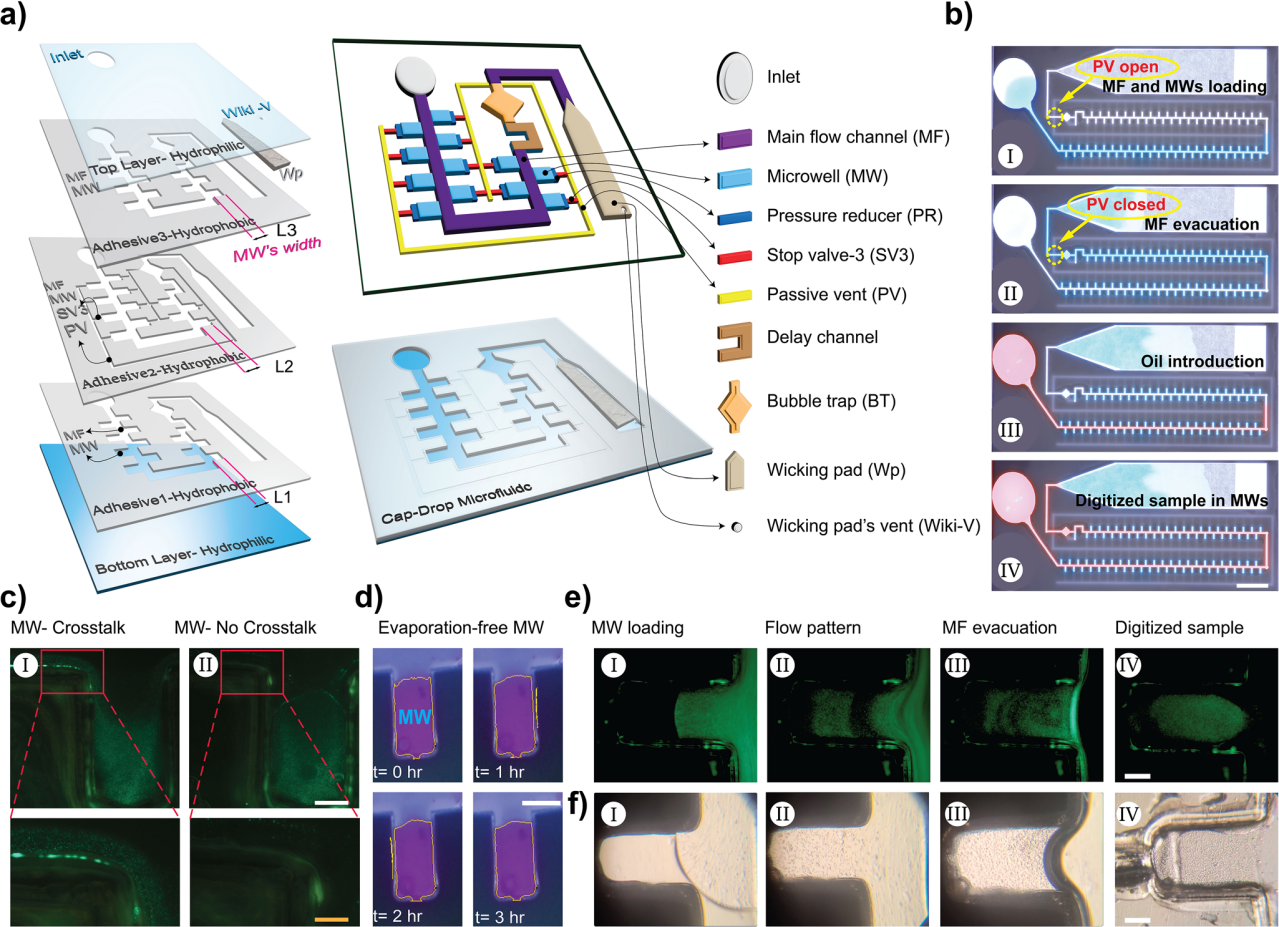
The Cap‐Drop design and working principle. a) Schematic overview of the Cap‐Drop, highlighting the assembly of individual layers and the capillary elements embedded within each layer. b) Optical images demonstrating the fluid progression through Cap‐Drop at different phases: (I) initial sample loading, (II) evacuation of the main flow channel (MF), (III) introduction of the immiscible phase, and (IV) formation of stationary droplets in microwells (MWs) (sample digitization). Scale bar: 7 mm. c) Fluorescent images showing (I) crosstalk between MWs, (II) elimination of crosstalk following the immiscible phase introduction. Scale bars: 140 and 350 µm, respectively. d) Stationary droplets confined in MWs with no observable evaporation. Scale bar: 300 µm. e) Fluorescent images of nanoparticle distribution, detailing: (I) MW loading, (II) flow patterns within MWs, (III) Evacuation, and (IV) final stationary droplet formation. Scale bar: 140 µm. f) Optical images showing Caco‐2 cells inside MWs, illustrating: (I) cell introduction, (II) flow patterns, (III) evacuation, and (IV) final positioning within MWs. Scale bar: 140 µm.

Cap‐Drop comprises five distinct layers (**Figure** **1**a), each with unique properties critical for controlling liquid flow through capillary elements. The bottom and top layers are made from hydrophilic polyethylene terephthalate (PET), providing the capillary force needed to drive the sample solution through the main flow channel (MF) and MWs. The bottom layer is pattern‐free and simply provides capillary force, while the top layer includes patterns for the inlet and the wicking pad vent (Wiki‐V), the only points exposed to open air for sample injection and air exit during the loading phase. Adhesives 1, 2, and 3 are hydrophobic, double‐sided adhesives with different patterns, but all share the MF and MWs. Adhesive 2 has an additional pattern of SVs and PVs and is sandwiched between adhesives 1 and 3. As a result, these elements are isolated from the top and bottom layers, making them fully hydrophobic and preventing the sample solution from entering. MWs length is the same in both Adhesive 1 and 2 (L1 = L2), but it is shorter in Adhesive 3 to create a small step called a PR (L3 < L2), which locally reduce capillary pressure before each SV. This helps improve SV stability and minimizes the risk of failure during sample loading. To ensure ease of fabrication, the patterning of layers should start with Adhesive 1, followed by Adhesives 2 and 3, and then the top layer. Once the patterning of Adhesive 1 is complete, the next layer is immediately attached over it. The CO2 laser then proceeds to pattern Adhesive 2, and the process continues sequentially through the third, fourth, and fifth layers. A wicking pad (Wp) is inserted into the chip prior to the assembly of the bottom layer, providing a secondary capillary source needed to remove excess sample from the MF. With this innovative fabrication process, by the time the laser finishes patterning all the layers, they are already pre‐aligned and bonded together, removing the need for any alignment.

Cap‐Drop operates by leveraging capillary forces to autonomously manipulate fluids and to generate and position discrete droplets. Once the sample solution is introduced to the Cap‐Drop's inlet, it moves through the MF and MWs (Figure 1b‐I), driven by the capillary forces generated by the hydrophilic layers. As the sample enters MWs, air is vented out through PV, which remains open during the loading phase. Once all MWs are filled and the sample solution reaches the Wp, the loading phase is complete, and PV automatically closes, trapping the sample within the MF and securing droplets in each MW (Figure 1b‐II). Following this, the MF is evacuated via the Wp's capillary force (Figure 1b‐III), digitizing sample solution within MWs. However, as depicted in Figure 1c‐I, crosstalk between MWs was observed during initial testing, where fluorescent nanoparticles were seen migrating between adjacent MWs through a thin residual aqueous film remaining on the MF walls after sample evacuation. The presence of these unintended fluid connections disrupts the system's goal of generating discrete droplets that can serve as separate reaction chambers for various applications. To address this issue, an immiscible oil phase is introduced to the Cap‐Drop inlet, filling the MF (Figure 1b‐IV) and forcing air out through Wiki‐V. This step eliminates crosstalk (Figure 1c‐II) and significantly reduces evaporation of the aqueous samples within MWs (Figure 1d), as they are sealed from ambient air. The oil moves through the MF via capillary force, facilitated by the MF's vertical walls made from oil‐friendly, non‐polar material that forms a low contact angle with the oil (Table S1 and Figure S4, Supporting Information). To enhance oil's flow rate through MF, a critical micelle concentration of Span 20 is added to lower the oil's contact angle with the MF's surfaces, further enhancing capillary forces and accelerating oil movement. The performance of the Cap‐Drop in digitizing samples and eliminating crosstalk between MWs is seen in Movie S1 (Supporting Information). The flow pattern within MWs in Cap‐Drop is demonstrated for microparticles and human cells in Figure 1e,f, respectively. Variations in viscosity, concentration, size, and mass between cells and microparticles lead to distinct flow behaviors within MWs. These flow properties should be studied for each target sample to help optimize the Cap‐Drop, making it a versatile microfluidic system.

### 2.2 Capillary Elements

The Cap‐Drop employs a variety of innovative capillary elements, compatible with the laser cutting fabrication process, to streamline and enhance the three essential phases for sample digitization: loading the MF and MWs with sample solution, evacuating the MF, and filling the MF with oil (Movie S1, Supporting Information). Successful MF and MW loading requires three key factors: providing adequate capillary force to drive the sample into the MF and MWs, creating an air exit at the end of each MW to ensure bubble‐free filling, and stopping the flow at the end of MWs before it reaches PV. The capillary force is generated by the hydrophilic PET used in the top and bottom layers, satisfying the first requirement. The PV addresses the second need by providing a pathway for air to exit. The SVs fulfill the third requirement by halting the sample flow at the end of MWs, preventing it from entering the PVs. Without reliable SVs, the sample would fill the PVs, leading to failed digitization even after MF evacuation, as MWs would remain interconnected through the PVs. Thus, the performance of the PVs in Cap‐Drop is tightly linked to the effectiveness of the SVs.

#### 2.2.1 Stop Valve and Pressure Reducer Elements

To create an effective SV, minimizing or even eliminating the capillary force in the designated area is essential. In the initial design, termed SV1, only one adhesive layer (Figure S1a‐I, Supporting Information) was used in the middle of the Cap‐Drop‐SV1. The narrow channels at the end of MWs, connecting MWs to the PV, were intended to function as SV1. However, both experimental results and two‐phase fluid flow analysis indicated that simply narrowing the channels was not effective (Figure S1b‐I,c‐I, Supporting Information). According to the Yang‐Laplace equation,^[^ ^46^ ^]^ while narrowing the channel reduces the capillary force, a positive capillary force remains active due to the hydrophilic nature of the top and bottom surfaces of the SV1, allowing the sample to continue moving, albeit more slowly, rather than stopping entirely. In Cap‐Drop‐SV2, adhesive 1 (Figure S1a‐II, Supporting Information) was added beneath adhesive 2, covering the bottom layers of both the SVs and PV with a hydrophobic material. However, this modification also failed to stop the sample solution, as SV2 burst without offering significant resistance (Figure S1b‐II,c‐II, Supporting Information). To improve the SV's effectiveness in SV3, adhesive 3 (Figure S1a‐III, Supporting Information) was added on top of adhesive 2, creating a barrier between the SV3 and the hydrophilic PET used as the topmost layer of the Cap‐Drop‐SV3 (simplified to Cap‐Drop). This configuration proved successful, with all four sides of the SV channel made from hydrophobic adhesive (Figure S1b‐III,c‐III and Movie S2, Supporting Information).

However, despite the hydrophobic nature of the materials, some sample solutions demonstrated a contact angle slightly greater than 90° (Table S2, Supporting Information), which, while reducing the capillary forces significantly, was insufficient to fully resist the flow under increased pressure, occasionally causing the SV to burst.

**Figure 2:**
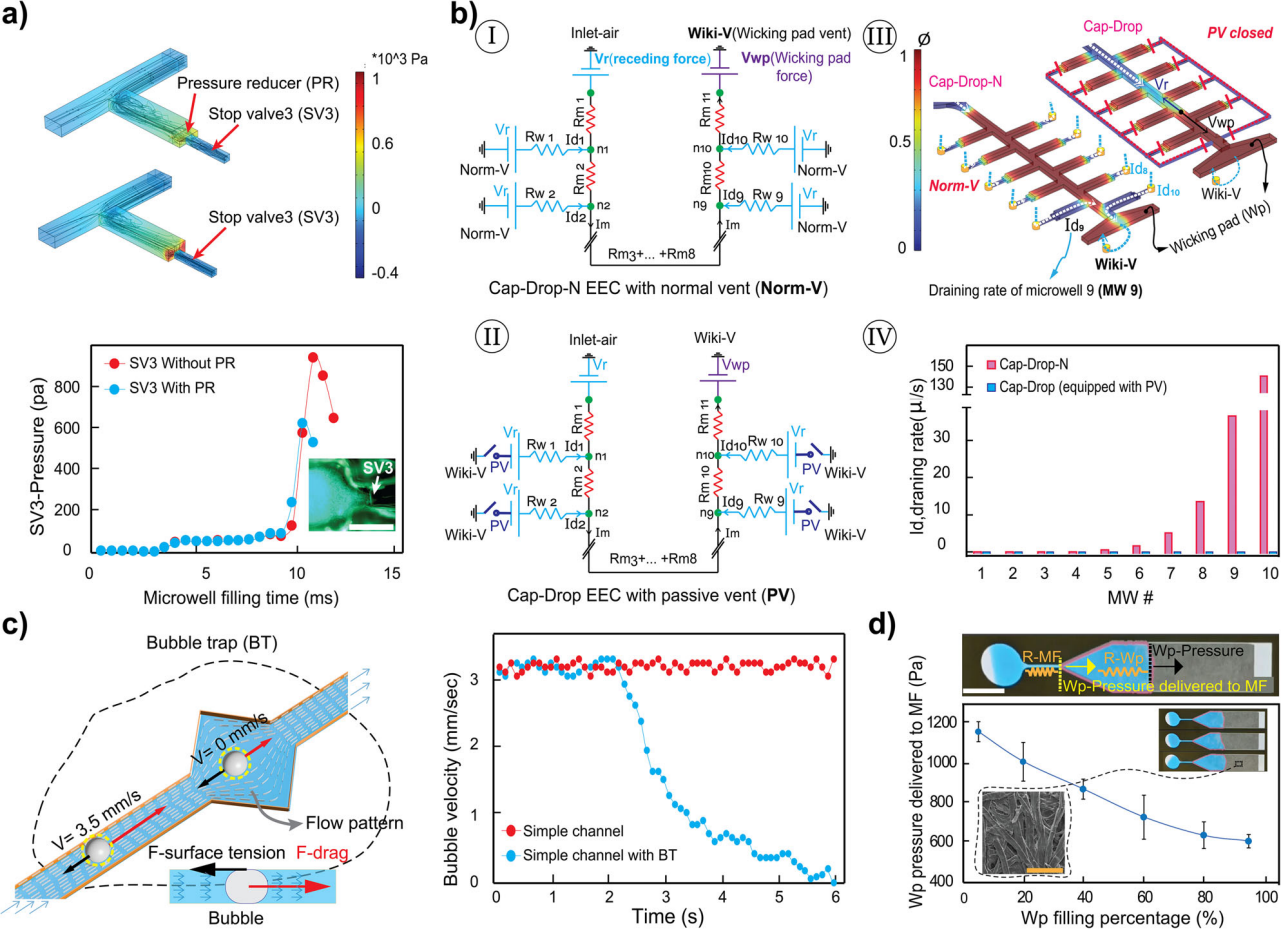
Performance analysis of Cap‐Drop's capillary elements. a) Two‐phase fluid flow simulation using the level set module in COMSOL Multiphysics, showing the effect of pressure reducer (PR) on the pressure encountered by the stop valve version 3 (SV3) at initial contact. Regions where ϕ > 0.5, indicating areas filled with water, are displayed in red, while areas where ϕ < 0.5, indicating air, are shown in blue. Scale bar: 400 µm. b) Performance comparison of Cap‐Drop‐N with normal vent (Norm‐V) and Cap‐Drop with passive vent (PV). (I) Equivalent electrical circuit (EEC) for Cap‐Drop‐N. (II) EEC for Cap‐Drop equipped with PV. (III) COMSOL Multiphysics simulation comparing MF evacuation between Cap‐Drop‐N and Cap‐Drop with PV. (IV) MW draining profiles in Cap‐Drop‐N and Cap‐Drop with PV. c) COMSOL Multiphysics simulation of the bubble trap (BT) capturing bubbles in contrast to a simple channel without BT. d) Power delivered by the wicking pad (Wp) to the MF, with power varying as a function of Wp's filling percentage. Scale bars: 1 cm and 100 µm.

To enhance the reliability of SV3, the length of MWs in adhesive 3 was shortened by 20% compared to adhesive 2 (L3 < L2; Figure 1a), introducing a small step called a PR before each SV3. As shown in two‐phase fluid flow simulations using COMSOL Multiphysics (**Figure** **2**a), the PR effectively reduces the pressure exerted on the SV3 when the sample solution first contacts it. Although the inclusion of a PR is not strictly essential, it is recommended as a precautionary measure to mitigate the risk of valve failure, as the malfunction of a single valve could compromise the entire operation of the Cap‐Drop. Since SV3 was determined to be the optimal configuration, it is referred to as SV in previous sections and throughout the remainder of this work.

#### 2.2.2 Passive Vent and Delay Channel Elements

The PV network within Cap‐Drop is a critical design feature, connecting all MWs and providing a safe pathway for air to escape during MW loading (Figure 1a). Its importance is highlighted when compared to a simpler alternative, Cap‐Drop‐N, which uses normal vents (Norm‐Vs) in place of the PV network (details in Figure S2, Supporting Information). In Cap‐Drop‐N, each MW is equipped with a small hole (Norm‐V) that allows air to escape. While this design simplifies construction by requiring only one adhesive layer and eliminates the need for PV and SVs, it introduces significant risks of evaporation and contamination. These issues are particularly critical for the small liquid volumes handled in MWs (40 to 500 nL), where even minor evaporation is intolerable (Figure S2d, Supporting Information). Although Cap‐Drop‐N meets the criteria for successful loading of MF and MWs (Figure S2b‐I,c‐I, Supporting Information), it fails during the second step of the Cap‐Drop workflow—MF evacuation—as illustrated by its equivalent electrical circuit (EEC) in Figure 2b‐I and Movie S3 (Supporting Information).

For successful MF evacuation, the sample solution must reach the Wp, where its capillary force effectively evacuates the MF without draining MWs (Figure 1b‐II). EEC was designed to analyze the evacuation performance of Cap‐Drop‐N by considering all forces acting on the fluid and the resistance encountered during the evacuation phase. In Cap‐Drop‐N, a positive capillary pressure is generated by the Wp, which is calculated through image processing, as discussed in detail in the Wp section. While a negative pressure arises due to the receding contact angle caused by the hydrophilic layers during MF draining. Furthermore, the Norm‐V introduces air access to each MW, generating reverse pressure at the end of each MW. This reverse pressure is determined using the Yang‐Laplace equation (Equation S1, Supporting Information). In the designed EEC (Figure 2b‐I), the Wp and reverse pressure are represented as voltage sources (Vwp and Vr, respectively), while air access points, such as the inlet, Norm‐V, and Wiki‐V, are depicted as ground symbols. The flow rate (Q) corresponds to electrical current (I_d_), with MWs and MF represented as resistors (R_w_ and R_m_, respectively). EEC analysis for Cap‐Drop‐N revealed high drainage rates (I_d9_ and I_d10_) in MW9 and MW10 (Figure 2b‐IV), confirmed by COMSOL Multiphysics simulations and real experiments (Figure 2b‐II,III; Figure S2c‐II, Supporting Information). As a result, air entered the Wp through the Norm‐Vs in MW9 and MW10, disconnecting the Wp from the sample solution in the MF and leading to failed MF evacuation.

Accordingly, the need for a PV arises from the Cap‐Drop's workflow, where the vent at the end of each MW must remain open during the loading phase to allow air to escape from the chip. It must then close automatically as Cap‐Drop transitions from the loading phase to the MF evacuation phase. Closing the vents during the evacuation phase prevents air from re‐entering MWs, ensuring that drainage occurs only through the MF and not MWs. This critical function is enabled by the PV design (Figure 1a), in conjunction with the Wiki‐V located at the end of the Wp. The PV consists of a network of channels connecting all MWs, with its last branch linked to the MF, providing access to the Wiki‐V when it is not filled with sample solution (Figure 1b‐I). The PV remains open as long as it maintains access to the Wiki‐V and closes automatically once the sample solution reaches the last branch of the PV (Figure 1b‐II). A delay channel placed before the PV's last branch ensures sufficient time for all MWs to fill before the PV closes (Figure S3 and Movie S4, Supporting Information). The corresponding EEC for Cap‐Drop equipped with a PV is shown in Figure 2b‐II, where the PV is represented as an automatic cut‐off switch. During the loading phase, this switch remains closed, providing access to air (ground), while during the evacuation phase, the switch opens, cutting off air access. EEC analysis of Cap‐Drop revealed that no current flowed through MWs (Figure 2b‐III), a result confirmed by COMSOL Multiphysics simulations (Figure 2b‐IV) and experimental data (Figure 1a‐III), where the sample solution remained confined within MWs after MF evacuation. The successful incorporation of the PV and SVs not only meets all the criteria for successful loading and evacuation in Cap‐Drop but also eliminates risks of evaporation and contamination (Figure 1d). This improvement stems from replacing all Norm‐Vs at the end of MWs in Cap‐Drop‐N with the PV, which works in conjunction with the Wiki‐V to enhance Cap‐Drop's enclosed design. In the final step of the Cap‐Drop workflow, an immiscible oil phase is introduced to completely fill the MF under capillary flow (Figure 1b‐IV), ensuring full encapsulation and digitization of droplets within MWs.

#### 2.2.3 Bubble Trap Element

During the evacuation phase of the MF, air bubbles may potentially enter the system. This typically occurs through the inlet, especially as it starts draining. The likelihood of this depends on the sample's adhesion to the inlet surface and its physical properties. Air bubbles may also from via small gaps between adhesive layers caused by misalignment or minor fabrication issues. If a bubble forms, it may travel toward the end of the MF and create a gap between the Wp and the remaining sample solution, ultimately leading to unsuccessful evacuation of Cap‐Drop. To mitigate this risk, a BT was designed and positioned before the Wp. The BT operates based on Newton's second law, reducing the bubble's velocity to zero at its trapped position, thereby preventing it from reaching the Wp (Movie S5, Supporting Information). This is achieved by designing the BT with a wider width compared to the MF, which reduces the flow rate and decreases the drag force acting on the bubble (Equation S4, Supporting Information). Furthermore, the pentagonal shape of the BT induces a flow pattern around the bubble, stabilizing it in an equilibrium position. Surface tension between the bubble and the MF walls also plays a critical role in the BT's functionality, further immobilizing the bubble at the trap location (Figure 2c). It is noted that the BT has a finite capacity and may become saturated if the bubble volume exceeds its holding capacity. In such cases, the bubble could escape the trap and reach the Wp, potentially resulting in the failure of the Cap‐Drop. For example, when an air bubble introduced through Norm‐V in Cap‐Drop‐N exceeded the capacity of the BT, the chip was unable to complete the evacuation phase (Movie S3, Supporting Information).

#### 2.2.4 Wicking Pad Element

The microstructural characteristics of the fibrous Wp (Figure 2d), including its pore size, shape, and distribution, play a critical role in determining the pressure it exerts. Accurately quantifying this pressure range is crucial for validating both the equivalent EEC and numerical simulations. To achieve this, a simple experiment was conducted in which 86 µL of water was introduced into a system consisting of an inlet, a simple channel like the MF, and the Wp. The water absorption rate of the Wp was measured using a Python‐based image processing library (detailed in Movie S6, Supporting Information). This absorption rate corresponds to the flow rate (Qs) in the simple channel connecting the inlet to the Wp. With the flow rate and the hydraulic resistance of the simple channel (R_mf_) determined using Python and Equation S2 (Supporting Information),^[^ ^46^, ^47^ ^]^ the capillary pressure across the R_mf_ (ΔP) was calculated using the Hagen‐Poiseuille equation (Equation S3, Supporting Information).^[^ ^48^ ^]^ In this experiment, gravitational effects were neglected, and the inlet pressure applied to the simple channel was assumed to be zero. As a result, the calculated ΔP represents the pressure generated by the Wp and applied to the simple channel. As shown in Figure 2d, filling the Wp increases its resistance (R_wp_) within the EEC, thereby reducing the pressure transferred to the R_mf_, indicated by the yellow resistance. While the R_mf_ resistance remains constant in this experimental setup, it decreases dynamically in the Cap‐Drop as the MF drains in evacuation phase. This simultaneous reduction in both the pressure delivered to the MF by the Wp and the R_mf_ resistance during evacuation phase of Cap‐Drop causes a nearly constant evacuation rate.

### 2.3 Parameters Optimization

**Figure 3:**
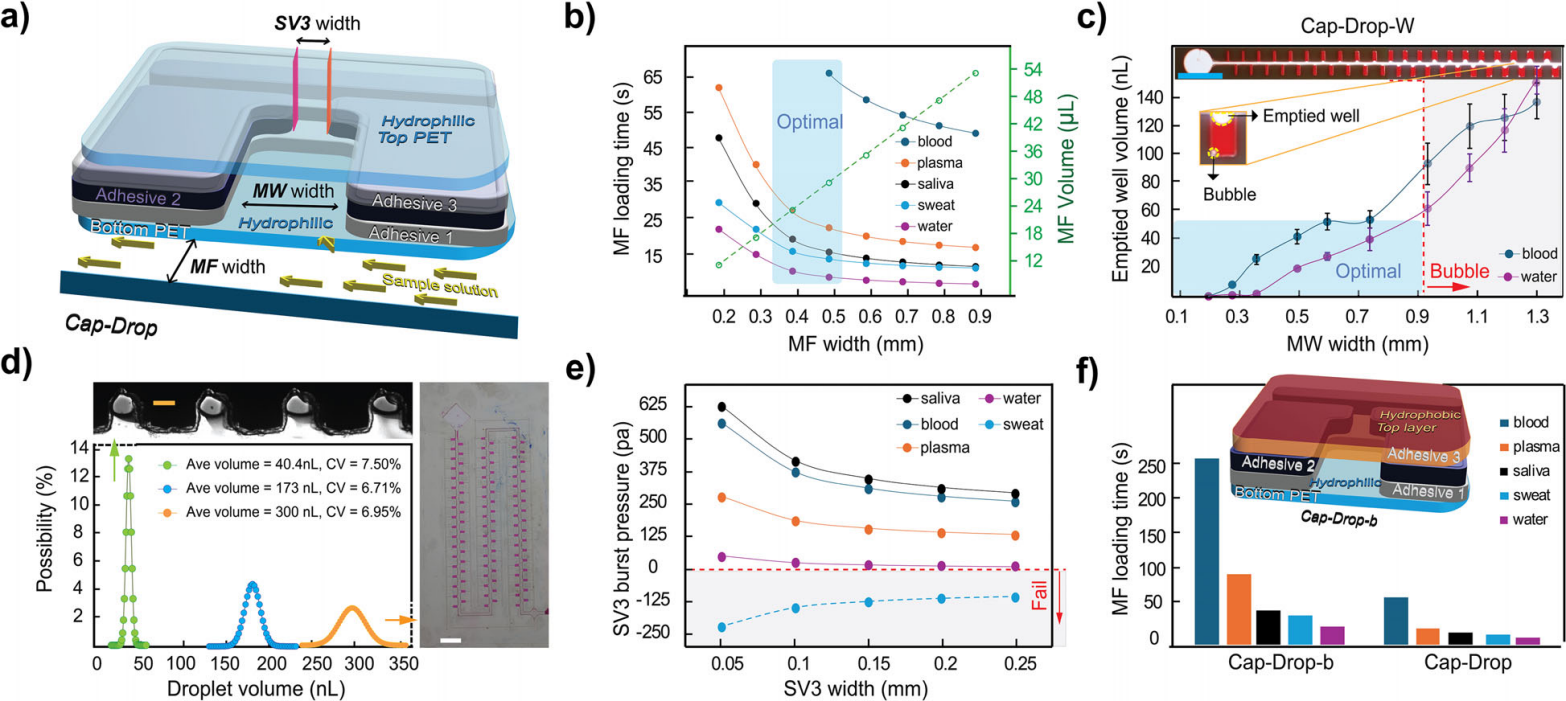
Parameter optimization for the Cap‐Drop. a) Schematic of the Cap‐Drop highlighting key parameters that were optimized. b) Influence of MF width on both MF loading time and MF volume tested with various biofluids. c) Impact of MW width on the emptied well volume and bubble formation at the MW's end. Scale bar: 3 cm. d) Normal distribution and uniformity of MW volumes for Cap‐Drop with different target volumes: 40, 150, and 300 nL. Scale bar: 5 cm and 500 µm. e) Burst pressure analysis of the SV tested with various biofluids and the effect of SV's width on pressure resistance. f) Comparing the loading times between the standard five‐layer Cap‐Drop and the four‐layer Cap‐Drop‐b variant.

The performance of the Cap‐Drop in droplet generation is influenced by the dimensions of its compartments and capillary elements (**Figure** **3**a), as well as the physical properties of the target biofluid. These critical aspects were thoroughly analyzed below.

#### 2.3.1 Optimizing the Main Flow Channel's Width

The MF dimensions play a pivotal role in influencing capillary pressure, hydraulic resistance, and overall fluid flow within the system. Using the Hagen‐Poiseuille equation (Equation S3, Supporting Information), the flow rate is calculated based on both capillary pressure and hydraulic resistance. The pressure in the MF, with its rectangular cross section, varies depending on the contact angles of the channel walls and is determined using the Young‐Laplace equation (Equation S1, Supporting Information). The resistance, computed via Equation S2 (Supporting Information), is dynamic and evolves as the fluid interface progresses through the MF, reflecting changes in flow conditions.

To model the flow rate within the MF as the fluid interface advances, the MF is divided into small segments, each with nearly constant resistance. The flow rate for each segment is calculated using the Hagen‐Poiseuille equation (Equation S3, Supporting Information) based on the interface's position. As shown in Figure S5 (Supporting Information), the capillary flow rate decreases as the channel fills, which is evident from the tracing of the air‐liquid interface. This decrease occurs because, while the pressure remains constant, the resistance increases with the volume of fluid in the MF, leading to a progressively lower flow rate.

To optimize the MF width, experiments were performed using a 20 cm MF with varying widths, tracking the loading time for each biofluid. Narrower channels increase resistance but reduce pressure due to the hydrophobic nature of the vertical walls. As shown in Figure 3b, the optimal width was identified by balancing loading time with the volume of sample retained in the MF, achieving an efficient compromise between these parameters for optimal performance.

#### 2.3.2 Optimizing the Microwell's Width

The uniformity of digitized sample volumes across more than 100 MWs in the Cap‐Drop is highly dependent on MW width. To investigate its impact on volume uniformity, we developed a variant called Cap‐Drop‐w, where MWs width gradually increases along the MF, with each width repeated four times, as illustrated in Figure 3c. This configuration was tested using two biofluids with differing viscosities: water and blood, representing low‐ and high‐viscosity fluids, respectively (Table S2, Supporting Information).

The effect of MWs width on the uniformity of digitized samples becomes evident during the MF evacuation phase when the MF and a portion of each MW are drained, forming a meniscus at the emptied MW. As demonstrated in Figure 3c, increasing the MW width leads to larger error bars in the emptied section, which compromises the uniformity of digitized sample volumes across MWs. Additionally, for MWs widths greater than 1 mm, bubbles form at the end of the MW due to the corner flow effect,^[^ ^46^ ^]^ disrupting the bubble‐free operation of Cap‐Drop. Recognizing the need for uniformity and a bubble‐free environment, we identified an optimized range for MWs width, highlighted by the blue rectangle in Figure 3c.

The precision of MW fabrication using a CO_2_ laser cutter also affects sample volume uniformity, in addition to the variation in emptied wells. By maintaining the MWs width between 0.5 and 0.7 mm within the optimized zone and varying other dimensions like the height and length of MWs, we developed three different Cap‐Drops. These devices successfully digitized sample to droplets with volumes of 40, 170, and 300 nL, achieving coefficients of variation (CV) of 6.31%, 6.71%, and 7.5%, respectively (Figure 3d). This demonstrates the system's versatility and flexibility in performance.

#### 2.3.3 Stop Valve Optimization

A simple rectangular channel was implemented as the SV to streamline the fabrication process of Cap‐Drop. Figure 3e presents the calculated burst pressure of the SV, derived from the Young‐Laplace equation (Equation S1, Supporting Information), which factors in the width, height, and contact angle between the SV walls and the biofluid. While the height of the SV is constrained by the thickness of adhesive 2, the contact angle is determined by the biofluid's properties. Consequently, the SV width becomes the sole adjustable parameter for optimization. As shown in Figure 3e, reducing the SV width increases the burst pressure, enhancing the SV's durability. However, the minimum achievable width in the Cap‐Drop is limited to 120 µm due to the precision constraints of the laser cutting process. Results indicate that the Cap‐Drop performs effectively with biofluids such as blood, plasma, saliva, and water. However, the findings also highlight the critical role of incorporating a PR to mitigate potential SV failure under lower pressures, particularly when working with low‐viscosity fluids like water. Notably, regardless of the SV width, the system struggled to prevent sweat from entering the PV, underscoring the need for further design adjustments when dealing with fluids of similar properties.

#### 2.3.4 Optimizing the Number of Layers

The use of hydrophilic sheets on both the top and bottom layers of Cap‐Drop generates strong capillary forces, enabling rapid sample loading. However, this design poses challenges for applications requiring precise and independent liquid deposition, such as delivering varying drug concentrations or nutrients into each MW via drop casting. In these scenarios, the hydrophilic surface causes the liquid to spread from one MW into the MF and adjacent MWs. To overcome this limitation, the Cap‐Drop‐b variant replaces the top hydrophilic layer with a hydrophobic adhesive. This modification prevents spreading and allows accurate drop‐casting directly into individual MWs. Drop‐casting of drugs or stimuli with varying concentrations is performed before sealing the chip, by dispensing droplets into each MW and allowing them to evaporate, leaving behind solid drug deposits. Once this step is completed, the bottom (hydrophilic) layer is attached, forming a fully enclosed microfluidic system. Upon sample introduction, the dried contents rehydrate, creating distinct local environments across the MW array. For scalable production, this workflow can be adapted to automated pipetting platforms.

**Figure 4:**
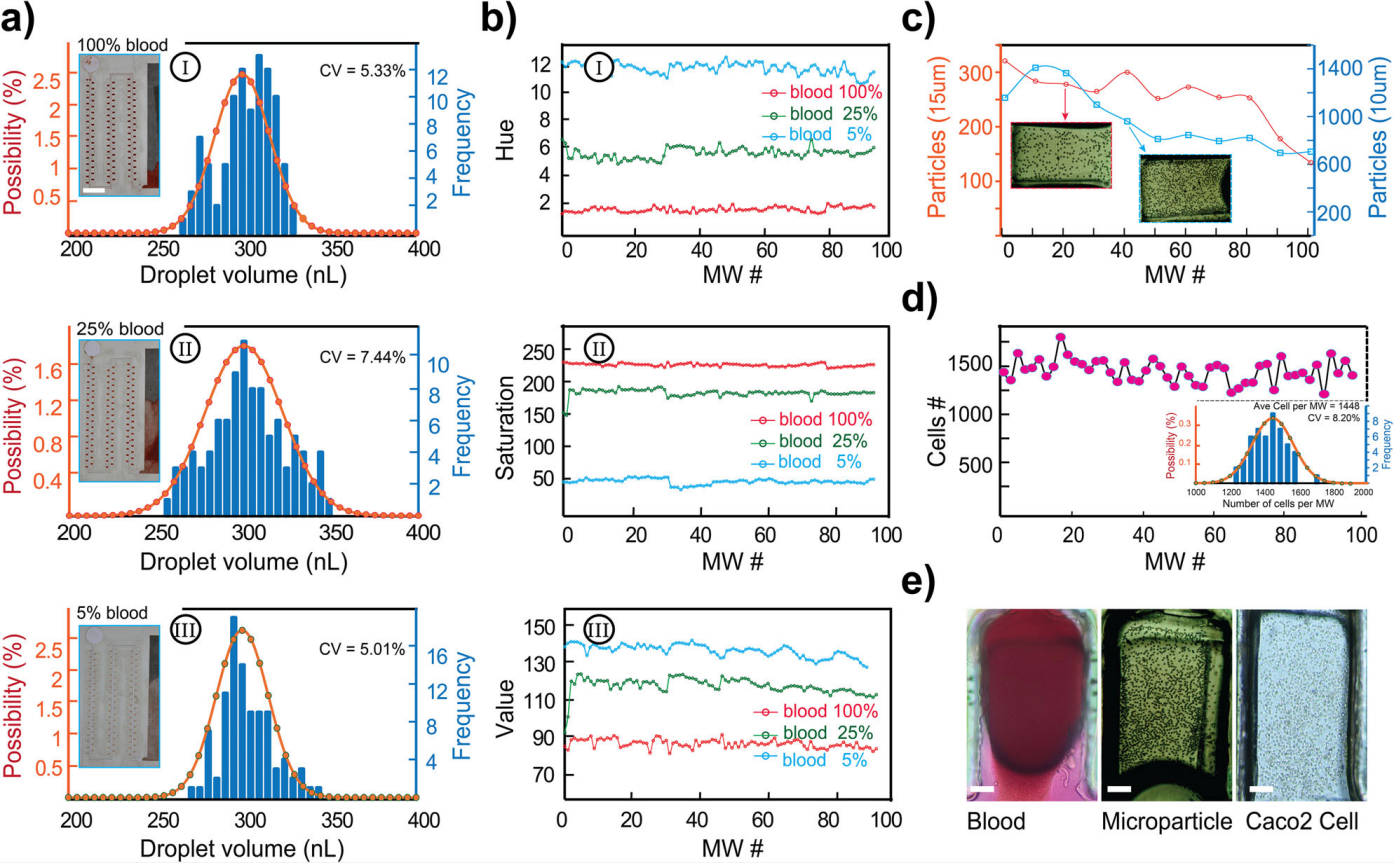
Particle distribution in Cap‐Drop. a) Normal distribution of digitized sample volumes with different whole blood concentrations: (I) 100% whole blood, (II) 25% whole blood diluted with serum, and (III) 5% whole blood diluted with serum. Scale bar: 6 cm. b) Distribution of red blood cells across all MWs, comparing hue, saturation, and value for whole blood concentrations of 100%, 25%, and 5%. c) Microparticle distribution comparison: 15 µm microparticles at a concentration of 1.35 × 10⁶ mL⁻¹ versus 10 µm microparticles at 4.55 × 10⁶ mL⁻¹ concentration. d) Distribution of Caco‐2 cells within a collagen hydrogel precursor, diluted in a medium with a 1:1:4:4 ratio of Sodium Hydroxide (NaOH), 4‐(2‐hydroxyethyl)‐1‐piperazineethanesulfonic acid (HEPES) buffer, collagen, and culture media, alongside the normal distribution of MW volumes. e) Visualization of digitized samples in the MWs: (I) Whole blood, (II) Microparticles, (III) Caco‐2 cells. Scale bar: 100 µm.

As shown in Figure 4f, the hydrophobic top layer increases the loading time for biofluids compared to the original Cap‐Drop. Additionally, it eliminates the need for adhesive layer 3, which previously acted as a barrier between the SV and the hydrophilic top layer. This reduces the MW height by 0.33% and decreases the sample volumes. The slower sample movement reduces the pressure on the SV, making the PR unnecessary. Cap‐Drop‐b simplifies the design to a four‐layer configuration while maintaining reliable performance.

### 2.4 Particle Distribution in Cap‐Drop

The earlier sections demonstrated the ability of the Cap‐Drop to uniformly trap volumes of diverse biofluids—including blood, plasma, saliva, and water—across MWs of varying sizes. However, further evaluations are needed to explore its adaptability and functionality with suspension‐based biofluids, such as cell suspensions, microparticles, or red blood cells in the whole blood. Future studies should focus on assessing the consistency of trapped volumes within each MW and the uniform distribution of suspended particles across more than 100 MWs, ensuring the system's suitability for broader biological and diagnostic applications.

A Cap‐Drop with MWs measuring 0.7 mm in width, 1.45 mm in length, and 0.3 mm in height was used to evaluate system performance. The estimated trapped volume in each MW was ≈300 nL. **Figure** **4**a shows the volume uniformity for blood samples at 100%, 25%, and 5% concentrations, diluted in serum. The CV for these concentrations were 5.33%, 7.44%, and 5.01%, respectively, indicating consistent volume distribution across MWs. This demonstrates the system's capability in managing none‐Newtonian samples with varying viscosities and densities. Due to the small size and high density of red blood cells, direct counting within each MW was impractical. Instead, color analysis was conducted across all MWs for each concentration OpenCV in Python. As illustrated in Figure 4b, the hue, saturation, and value were consistent across MWs, confirming a uniform distribution of red blood cells for all tested concentrations.

In a subsequent experiment, we analyzed the distribution of mono‐sized microparticles measuring 10 and 15 µm, at concentrations of 4.55 × 10⁶ and 1.35 × 10⁶ particles mL^−1^, respectively. Figure 4c displays the observed variations in particle counts across all MWs, primarily due to sedimentation and uneven sample distribution at the inlet. Notably, MWs at the end of the Cap‐Drop showed a significant reduction in particle counts due to sedimentation, while some MWs exhibited unexpectedly high counts, suggesting localized spikes in particle concentration. These findings indicate an uneven distribution of the sample solution, particularly at the inlet, highlighting challenges in achieving uniform particle distribution in the Cap‐Drop.

Caco‐2 cells were suspended uniformly in a medium containing 25% 4‐(2‐hydroxyethyl)‐1‐piperazineethanesulfonic acid (HEPES) and 75% collagen I, increasing the density to reduce sedimentation effects. As shown in Figure 4d, the distribution of cells across all MWs was highly uniform, with CV = 8.2%. This relatively low variation is mainly attributed to differences in trapped volumes within each MW, rather than uneven distribution of the sample. Figure 4e shows the MWs filled with the blood, microparticles, and Caco‐2 cells, illustrating the versatility of the system across various biofluids and suspended particles.

### 2.5 Culture of Caco‐2 Cells within Cap‐Drop‐Cell

**Figure 5:**
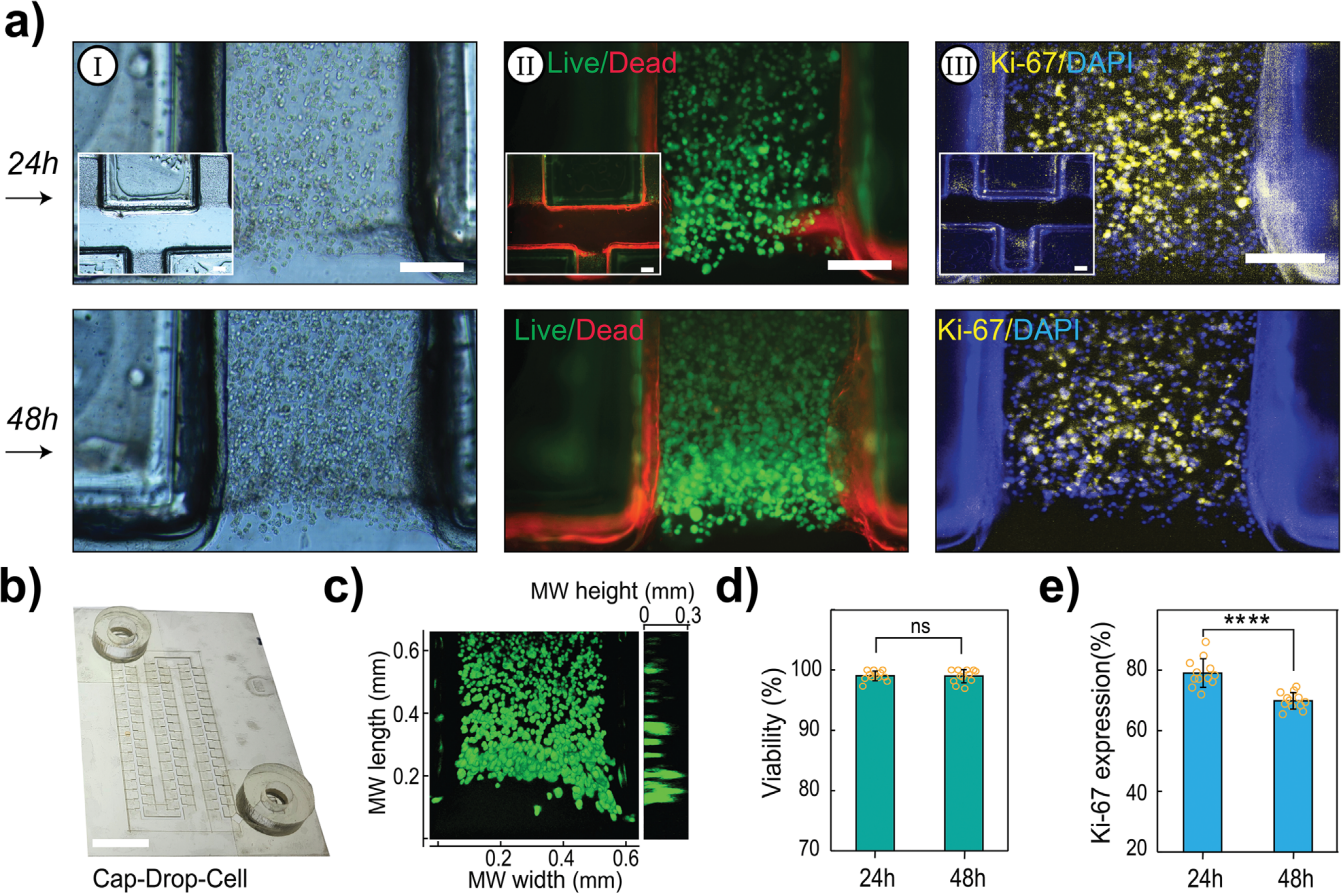
Viability and proliferation of Caco‐2 cells within Cap‐Drop‐Cell. a) (I) Brightfield images, (II) fluorescent LIVE/DEAD™ staining, and (III) immunofluorescence images of MKi67 expression for Caco‐2 cells encapsulated in Collagen I microgels and cultured for 48 h (scale bar: 200 µm). b) A photograph of the Cap‐Drop‐Cell device used for cell culture is presented (scale bar: 1 cm). c) Confocal microscopy and 3D reconstruction of the cultured cells are depicted, alongside. d) LIVE/DEAD assay results for cell viability. e) Quantification of cell proliferation based on MKi67 expression analyzed through immunofluorescence imaging. Statistical analyses were performed using one‐way ANOVA where ns stands for no statistical difference; and **** indicate P values <0.0001.

The Cap‐Drop‐Cell is an upgraded version of the Cap‐Drop, specifically engineered for cell culturing within MWs (**Figure** **5**b), in a 3D format (Figure 5c) with acceptable cell coverage across each MW over 48 h (Figure 5a‐I; Figure S6, Supporting Information). The design is fully compatible with cell tracking, live imaging, growth pattern analysis, and immunostaining, making it suitable for applications such as biocompatibility assessment, cell‐cell and cell‐matrix interaction studies, gene expression analysis, and drug testing. In the Cap‐Drop‐Cell, the MF evacuation is simplified using standard suction equipment commonly available in cell culture laboratories, eliminating the need for the Wp used in the original Cap‐Drop. Additionally, the design incorporates dual reservoirs to supply culture media during incubation (Figure 5b). A chip rocker facilitates continuous media flow through the MF, ensuring adequate aeration and nutrient delivery to MWs while efficiently removing waste materials. This setup supports optimal cell viability and enables extended culture periods, providing a versatile and user‐friendly platform for cell‐based assays.

In the Cap‐Drop microfluidic device, individual 3D cultures can be accessed using enzymatic (e.g., collagenase or trypsin) or mechanical methods for downstream analysis such as flow cytometry, RT‐qPCR, or single‐cell sequencing. As the Cap‐Drop device operates using capillary flow and does not require external pressure, we utilize pressure‐sensitive adhesive (PSA) to bond the top layer to the rest of the chip in a semi‐permanent manner. This allows the top layer to be securely attached during culture while enabling easy removal post‐fixation by applying minimal pressure during assembly. Peeling off the top layer grants access to all MWs and fixed reagents without disrupting the underlying structures, thus facilitating gentle and spatially resolved sample retrieval for post‐experimental analysis.

The results from the LIVE/DEAD assay indicated negligible cell viability in the Cap‐Drop‐Cell chips made with the material ARcare@93 049, which was identified as toxic (Figure S7, Supporting Information). In contrast, cells cultured in chips made from the bioinert material 3M‐9984 remained fully viable after 24 and 48 h of culture with showing 99.02% and 98.96% of live cells (Figure 5a‐II,d). Immunostaining for MKi67 revealed that 79.04% of cells were positive after 24 h of culture, and 69.89% were positive after 48 h, indicating robust cell proliferation (Figure 5a‐III,e). Cap‐Drop‐Cell microenvironment demonstrated excellent biocompatibility, supporting cell proliferation and viability within 3D Collagen I microgels for up to 48 h of incubation. This duration provides sufficient time for conducting most species reaction modeling and other related analyses.

In addition to collagen‐based hydrogels, the Cap‐Drop‐Cell is compatible with other biologically relevant materials such as Matrigel,^[^ ^49^ ^]^ fibrin,^[^ ^50^ ^]^ gelatin methacryloyl (GelMA),^[^ ^51^, ^52^ ^]^ and alginate^[^ ^53^ ^]^ which offer appropriate rheological properties for stable loading and confinement within MWs. For example, 5 wt.% GelMA—an increasingly common material in 3D cell culture—was successfully loaded into Cap‐Drop, demonstrating reliable performance at viscosities as high as ≈300 mPa·s. This represents a substantial increase over typical biofluids and highlights the platform's capacity to accommodate moderately viscous hydrogels used in advanced cell culture applications. Highly viscous, non‐shear‐thinning hydrogels may hinder performance due to flow resistance or swelling, which captures the trade‐off between viscosity and capillary flow rate. Materials like polyacrylamide (PAM) and poly(2‐hydroxyethyl methacrylate) (PHEMA),^[^ ^54^, ^55^ ^]^ and unmodified hyaluronic acid (HA)^[^ ^56^ ^]^ exemplify such limitations. Further details are provided in the Note S10 (Supporting Information).

To evaluate system performance under extended incubation, Cap‐Drop‐Cell chips were maintained at 37 °C in a humidified incubator for four days. Following 24 h of exposure, the media loading time increased from 15 to 48 s, likely due to the combined effects of elevated temperature and humidity. From day 2 to day 4, loading times increased more gradually to 55, 65, and 71 s, respectively, indicating consistent capillary performance over the full culture duration. Additional details on device dimensions and long‐term stability are provided in the Notes S11 and S12 (Supporting Information), respectively.

## 3 Conclusion

In this study, we successfully developed and optimized a capillary‐based droplet array microfluidic system for sample digitization from biofluids with diverse physical properties and particulate content (Figures 2 and 3). The Cap‐Drop and its variants demonstrate universal applicability and are fabricated using low‐cost, scalable materials, providing a practical alternative to systems reliant on glass or PDMS. The chip addresses major challenges in droplet microfluidics, including crosstalk, evaporation, and positioning, by reliably digitizing and trapping the sample within MWs and minimizing fluid migration and evaporation, even for nanoliter‐scale volumes (Figure 1c,d). Compared to vacuum‐based droplet arrays,^[^ ^28^, ^29^, ^30^, ^31^, ^32^, ^33^, ^34^ ^]^ the Cap‐Drop achieves significantly faster loading times, ranging from 10 to 60 s, depending on the biofluid, while Cap‐Drop‐b exhibits slightly longer loading times of up to 250 s (Figure 3f). The system's preprogrammed operation is achieved through the cooperative integration of capillary elements, including the PV, PR, SV3, Wiki‐V, Wp, delay channel, and BT. Each component performs its function sequentially without requiring external pumps, actuators, or manual interference, enabling fully autonomous operation. The design minimizes sensitivity to MW size and location, allowing variations from 0.2 to 0.6 mm (Figure 3c), while effective PV ventilation reduces the likelihood of bubble generation, a common issue in systems constructed with non‐gas‐permeable materials.^[^ ^41^ ^]^ The droplet volumes exhibit consistent uniformity, with a coefficient of variation (CV) below 7.5% (Figure 3d). Additionally, the self‐sealing mechanism of the PV, combined with the reliable performance of SV, ensures efficient evacuation of the MF, prevents air re‐entry, and suppresses evaporation. This performance is superior to the Cap‐Drop‐N variant, which lacks PV functionality and relies solely on Norm‐Vs (Figure S2, Supporting Information).

For applications requiring drop casting in individual MWs, such as throughput drug screening,^[^ ^2^ ^]^ antibiotic susceptibility testing,^[^ ^57^ ^]^ toxicity assays,^[^ ^58^ ^]^ and biochemical and biological analyses,^[^ ^59^, ^60^ ^]^ the Cap‐Drop‐b was developed. This variant reduces the number of layers compared to Cap‐Drop, decreases droplet volume by 30%, and incorporates MWs with hydrophobic bottoms to retain droplets during the drop‐casting process. Demonstrating the device's versatility, the Cap‐Drop‐Cell was introduced by eliminating the need for a WP and integrating two reservoirs to enable media replacement within the MF, ensuring cells receive essential nutrients during extended culture periods. Successful Caco‐2 cell proliferation and viability over 48 h within Cap‐Drop‐Cell highlights the device's biocompatibility and serves as a proof of concept (Figure 5). Cells were distributed across 101 MWs with a CV of 8.2%, demonstrating excellent uniformity (Figure 4d). Furthermore, Cap‐Drop‐Cell facilitated the precise and uniform distribution of cell‐laden hydrogel within MWs, yielding consistent microgel constructs. These constructs offer significant advantages for diverse applications, including spheroid studies,^[^ ^61^ ^]^ cancer invasion modeling,^[^ ^26^ ^]^ drug absorption analysis,^[^ ^62^ ^]^ and pathogenic infection modeling.^[^ ^63^ ^]^ A key innovation of Cap‐Drop‐Cell is its ability to easily remove residual hydrogel from the main channel after distribution into MWs, streamlining subsequent experimental workflows, such as media supplementation with toxins, drugs, or pathogens for targeted studies. Future optimizations will focus on minimizing cellular waste and enhancing nutrient delivery to extend cell viability for long‐term studies, while enabling throughput drug testing by drop casting varying drug doses into specific MWs before cell and media loading. Additionally, stepwise hydrogel introduction could create gradient microgels with distinct layers of varying density and composition, enhancing the Cap‐Drop‐Cell system's utility for replicating tumor microenvironments—a key direction for the next phase of this study.

## 4 Experimental Section

### Cap‐Drop fabrication

Cellulose paper (CFP42‐457) sourced from SterliTech, Inc. (USA) was used as the Wp. The flexible PET layer (9984 diagnostic microfluidic surfactant‐free medical hydrophilic film) was supplied by 3 m (USA). Pressure‐sensitive adhesive (PSA) sheets, including ARcare@8939 (a polyester film with double‐sided medical pressure‐sensitive adhesive), ARcare@90106NB (a flexible clear plastic film coated on both sides with medical‐grade pressure‐sensitive adhesive), and ARcare@93 049 (a diagnostic microfluidic surfactant‐free medical film), were provided by Adhesives Research, Inc. (USA). Span 20 (CAS# 1338‐39‐2), a co‐surfactant, was obtained from Sigma Aldrich. A laser cutter (Trotec Speedy 360 FLEXX 80 W CO_2_ and 30 W) was used to pattern microfluidic networks within the PSA layers, with ARcare@8939 containing fluorescence and ARcare@90106NB being fluorescence‐free. Additional PSA types with different heights and properties could also be used as alternatives. The fiber sheets were laser‐cut to fit the outlet, enabling efficient MF drainage and isolation of MWs. For cell culture applications, the Cap‐Drop version utilized 9984 hydrophilic PET as both the top and bottom layers due to its biocompatibility, whereas ARcare@93 049 was found to induce cell apoptosis. All adhesive layers, as shown in Figure 1a, were fabricated using ARcare@90106NB (fluorescence‐free), resulting in the MF and MWs height of 0.3 mm. To ensure smooth and stable channels with no PSA residue inside the microchannels, laser parameters were optimized for power (80), speed (70), frequency (5000 Hz), and two cutting passes.

### Numerical Simulation

The sample digitization workflow of the Cap‐Drop was simulated using COMSOL Multiphysics 6.1 software. The laminar two‐phase fluid flow was modeled in a 3D network using unsteady incompressible Navier‐Stokes equations along with Level Set variable equations.^[^ ^64^, ^65^ ^]^ Air pressure boundary conditions were applied to the inlet reservoir and various vent designs. To incorporate surface tension forces in the momentum equations, the static contact angle of the microchannel walls (top, bottom, left, and right) was considered. The necessary data for the simulation, including droplet contact angles, surface tension, and viscosity of different biofluids, are detailed in Tables S1 and S2 (Supporting Information).

### Particle Counting

Polystyrene microparticles of two sizes, (10 and 15 µm), were dispersed in deionized water to create stock solutions with concentrations of 4.55 × 10⁶ and 1.35 × 10⁶ particles mL^−1^, respectively, for the experiments. These stock solutions were prepared by carefully adding the microparticles to DI water and vortexing the mixture. The dilutions were then introduced into the Cap‐Drop for testing. After filling the chambers, mineral oil was introduced into the inlet using a capillary force to flush the microparticles from the main channel, effectively isolating the chambers. Once the MF was filled with mineral oil, each MW was imaged using a microscope to count the number of microparticles present. Python was used to estimate the hue saturation value of each microwell when whole blood was diluted to 100%, 25%, and 5% with serum. Ethics approval for working with blood was granted under REB number #REB16‐1147, issued by the University of Calgary Research Ethics Board.

### UV Sterilization for Microfluidic Devices

The assembled Cap‐Drop‐Cell were placed inside sterile Petri dishes and exposed to UV‐C light (254 nm) for 40 min using a UV sterilizer (UVP Cross‐linker ‐Analytikjena model, power setting 50). Following UV exposure, the Petri dishes were immediately sealed inside the sterilizer and then transferred to a cell culture hood, where they remained until use. This sterilization approach avoids heat, moisture, and chemical residues, making it well‐suited for preserving the structural and adhesive integrity of the PET and PSA layers used in Cap‐Drop‐Cell fabrication.

### Cell Culture

Caco‐2 cells (ATCC HTB‐37, human colorectal adenocarcinoma) were cultured in Eagle's Minimum Essential Medium (EMEM) supplemented with 10% (v/v) fetal bovine serum (FBS; ThermoFisher Scientific A5256701), 1% (v/v) MEM non‐essential amino acids (Sigma RNBJ8366), 1% (v/v) GlutaMAX (Gibco 35050–061), and 1% (v/v) penicillin/streptomycin (Sigma P4333). To optimize biocompatibility, chips were fabricated from two different hydrophilic materials (Figure S7, Supporting Information), and LIVE/DEAD assays were performed to determine the optimal material for further experiments. Cell densities of 100000, 150000, and 300000 cells were prepared following careful trypsinization and counting, and then introduced into the chips. A suspension of 150000 cells in (45 µL) of rat Collagen I solution (R&D Systems 3447‐020‐01), mixed with NaHCO₃ solution (37 g L^−1^; Sigma S5761) and HEPES buffer (15 630 080) in a 4:1:1 ratio, was introduced into each chip for subsequent experiments. For real‐time monitoring and live‐cell proliferation assessment, cells were treated with CellTracker CM‐Dil Dye (Invitrogen C7000), followed by two phosphate buffer saline (PBS) rinses prior to introduce into the chip. After introduction, cells homogeneously filled all >100 MWs, while those in the MF were removed using a vacuum pump at the chip outlet. The chips were incubated at 37 °C for 30 min to allow collagen hydrogel cross‐linking. Complete media was then added to the inlets and outlets, and the chips were incubated in a 37 °C humidified incubator with 5% CO₂.

### LIVE/DEAD Assay

To assess cell viability in the device, a LIVE/DEAD assay was performed. A mixture of calcein AM and ethidium homodimer (Invitrogen R37601) was introduced into the MF and incubated for 30 min at room temperature. Images from three different regions of the chips were captured at 24, and 48 h intervals. Image analysis using ImageJ software was performed, and cell viability was calculated after normalization.

### Immunostaining

Cells were fixed with a 3.7% (wt/v) paraformaldehyde solution, followed by two 5 min PBS washes. The cells were then permeabilized using 0.3% (v/v) Triton X‐100 permeabilization buffer (Sigma P1002116294) for 15 min, followed by two rinses with a washing solution composed of 2% (v/v) FBS. To block non‐specific binding, cells were treated with a blocking solution made of 2% (v/v) FBS, 2% (wt/v) bovine serum albumin (BSA; Sigma 810 533), and 0.1% (v/v) Tween 20 (Sigma P9416) for 45 min. Primary antibody against MKi67 (Sigma AB9260) diluted 1:50 in blocking solution was added, and the cells were incubated overnight at 4 °C. The chips were rinsed twice with the washing solution and treated with Alexa Fluor 647 goat anti‐mouse (Invitrogen A‐21241, 1:500 dilution), followed by a 1 h incubation at 37 °C. The samples were rinsed twice more with washing solution, stained with DAPI (Invitrogen D21490), and rinsed twice with PBS. Fluorescent images were analyzed using ImageJ software, and the ratio of proliferating cells was calculated at three different time points.

### Statistical Analysis

All experiments were performed with multiple replicates, as detailed in the figure captions. The data were expressed as mean ± SD, and statistical analysis was carried out using one‐way ANOVA followed by Tukey's post hoc test in GraphPad Prism v9.4.0.673. Comparisons between two groups were made using the unpaired t‐test in the same software, with a significance threshold set at *P* < 0.05.

## Conflict of Interest

The authors declare no conflict of interest.

## Author Contributions

P.J. and A.S.N. performed project conceptualization and design. P.J. performed device design, fabrication, experiments, data acquisition, analysis, and visualization. B.Z. and P.J. performed cell culture experiments. A.Z. and P.J. performed COMSOL simulations. P.J. and M.H. performed nanoparticle experiments. P.J., B.Z., S.A., and A.S.N. performed manuscript wrote the original draft. P.J., S.A., B.Z., M.V., A.Z., R.S., M.F., H.H., and A.S.N. performed manuscript editing and review. A.S.N. performed supervision and project guidance.

## Supporting information



Supporting Information

Supplemental Movie 1

Supplemental Movie 2

Supplemental Movie 3

Supplemental Movie 4

Supplemental Movie 5

Supplemental Movie 6

## Data Availability

The data that support the findings of this study are available from the corresponding author upon reasonable request.
