# Supplementary material for: Cap‐Drop: A Pre‐Programmed, Self‐Powered Capillary Microfluidic System for Passive Droplet Generation and 3D Cell Culture Modeling
Source: Small. 2025 May 22;21(25):2411997. doi: 10.1002/smll.202411997 (PMC12199121; doi:10.1002/smll.202411997)
Supplement: Supplementary file 1 — Supporting Information [file SMLL-21-2411997-s003.docx]

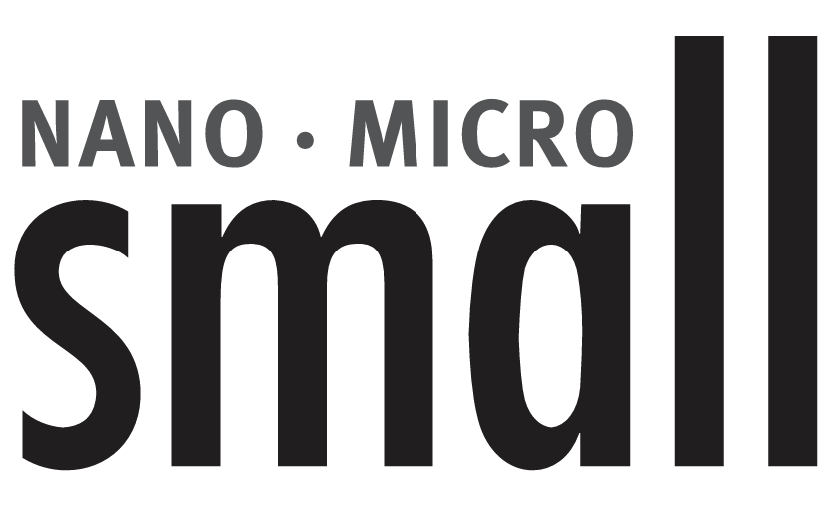


Supporting Information for

**Cap-Drop: A Pre-Programmed, Self-Powered Capillary Microfluidic System for Passive Droplet Generation and 3D Cell Culture Modeling**

**P. Jalali** *et al.*

Corresponding author: A. Sanati-Nezhad, [*asanatinezhad@ucalgary.ca*](mailto:asanatinezhad@ucalgary.ca)

**The PDF file includes:**

- Supplementary Notes
- Supplementary Figures S1 to S7
- Supplementary Tables S1 to S3
- References

**Table of Contents**

[**Note S1. Stop valves** 3](#_Toc194926434)

[**Note S2. Cap-Drop-N** 4](#_Toc194926435)

[**Note S3. Delay channel** 5](#_Toc194926436)

[**Note S4. Contact angle measurements of the water and different oils on different material layers of Cap-Drop** 6](#_Toc194926437)

[**Note S5. Effect of biofluid properties on fluid flow inside Cap-Drop** 7](#_Toc194926438)

[**Note S6. Determining the stop valve’s and Wicking pad’s capillary pressure** 8](#_Toc194926439)

[**Note. S8 Cell distribution and coverage within MWs** 9](#_Toc194926440)

[**Note S9: Biocompatibility evaluation of hydrophilic PETs** 10](#_Toc194926441)

[**Note S10: Viscosity constraints and hydrogel compatibility in capillary-driven systems** 10](#_Toc194926442)

[**Note S11. Dimensional Parameters of the Cap-Drop-Cell Device** 11](#_Toc194926443)

[**Note S12. Material stability and storage considerations for Cap-Drop** 11](#_Toc194926444)

[**Note S13. Movies** 11](#_Toc194926445)

[**Note S14. Reporting summary** 12](#_Toc194926446)

[**Note S14.1 Data collection:** 12](#_Toc194926447)

[**Note S14.2 Data analysis** 12](#_Toc194926448)

[**Note S14.3 Sample size and replication** 12](#_Toc194926449)

[**Note S14.4 Randomization** 13](#_Toc194926450)

[**Note S14.5 Blinding** 13](#_Toc194926451)

[**Note S14.6 Data exclusion** 13](#_Toc194926452)

[**Note S14.7 Antibodies** 13](#_Toc194926453)

[**Note S14.8 cell line source** 14](#_Toc194926454)

[**Note S14.9 Ethics oversight** 14](#_Toc194926455)

**References** ………………………………………………………………………………… 14

# **Note S1. Stop valves**

The layer configurations of different stop valve (SV) designs are shown in **Figure S1**. All adhesive layers are hydrophobic, while the top and bottom layers are hydrophilic in specific configurations. SV-1, SV-2, and SV-3 have two, one, and zero hydrophilic sides, respectively. Simulation and experimental results (Figure S1.b, c) show that SV-1 and SV-2 fail to stop flow at the microwell (MW) ends, while SV-3, with fully hydrophobic walls, successfully halts the flow.

In the Cap-Drop-b configuration, where the top layer is hydrophobic, SV-2 (Figure S1.a.ii) behaves like SV-3, with all walls being hydrophobic. This results in slower filling of the main flow (MF) channel and MW chambers due to the single hydrophilic bottom layer. Despite this, the hydrophobic top layer enables efficient drop-casting, making it ideal for applications such as drug testing and cell culture.





**Figure S1.** Stop Valves (SVs) performance. **a** Schematic diagrams of the stop valves (SVs), showing the number of layers in: i) SV-1, ii) SV-2, iii) SV-3. **b** Simulation of valve performance in stopping fluid flow at the end of the microwell (MW) during the main flow (MF) channel and MW loading, using the Level Set module in COMSOL. Areas with ϕ > 0.5 are displayed in red, representing regions occupied by water, while areas with ϕ < 0.5 are shown in blue, indicating areas occupied by air. "F" denotes the point of failure for each valve. **c** Visual representation of i) SV-1, ii) SV-2, iii) SV-3, focusing on the valves’ ability to stop fluid flow at the end of the MW during MF and MW loading.

# **Note S2. Cap-Drop-N**

Cap-Drop-N incorporates a small hole, termed the Normal Vent (Norm-V), at the end of each MW, allowing air to exit the chip during MW loading (**Figure S2**). This design ensures bubble-free loading of MWs and eliminates the need for SVs, as confirmed by both simulations and experiments. The Norm-V design effectively stops flow at the MW ends (Figure S2.b.i and S2.c.i). However, when the wicking pad (Wp) starts draining the MF channel, air re-enters through the Norm-Vs of MWs nearest to the Wp, causing a disconnect between the MF flow and the Wp. This leads to a pause in MF drainage (Figure S2.b.ii and S2.c.ii). Additionally, the presence of Norm-Vs results in a high evaporation rate inside the MWs, which becomes a critical issue when handling nanoliter-scale volumes.





**Figure S2.** The Cap-Drop-N performance**. a** The schematic highlights the small hole at the normal vent (Norm-V). **b** Simulations demonstrate: (I) successful, bubble-free loading of the MWs and effective flow stoppage at the MW ends without requiring a stop valve, and (II) unsuccessful evacuation of the MF channel due to air re-entering through the Norm-V. The Level Set module in COMSOL was used for simulations, with water-occupied regions (ϕ > 0.5) shown in red and air-occupied spaces (ϕ < 0.5) shown in blue. **c** Experimental results confirm: (I) successful bubble-free MW loading and effective flow stoppage at the MW ends without a stop valve, and (II) unsuccessful MF channel evacuation, consistent with simulation results. Scale bar: 2 mm. **d** The Norm-V design also leads to evaporation within MWs, as visualized in experimental tests. Scale bar: 500 µm.

# **Note S3. Delay channel**

Some MWs do not fill sequentially but rather randomly, which is due to the presence of wall roughness. This roughness effect is observable when using MWs with a width of less than 0.3 mm (Movie S4). The inclusion of a delay channel ensures that all MWs have adequate time to fill completely before the sample solution seals the primary vent (PV) (Figure S3.b). If the sample solution reaches the last branch of the PV (Figure S3.c) before all MWs are fully loaded, the closure of the PV traps air inside the unfilled MWs, preventing them from completing the loading phase.**_­­_**

**_
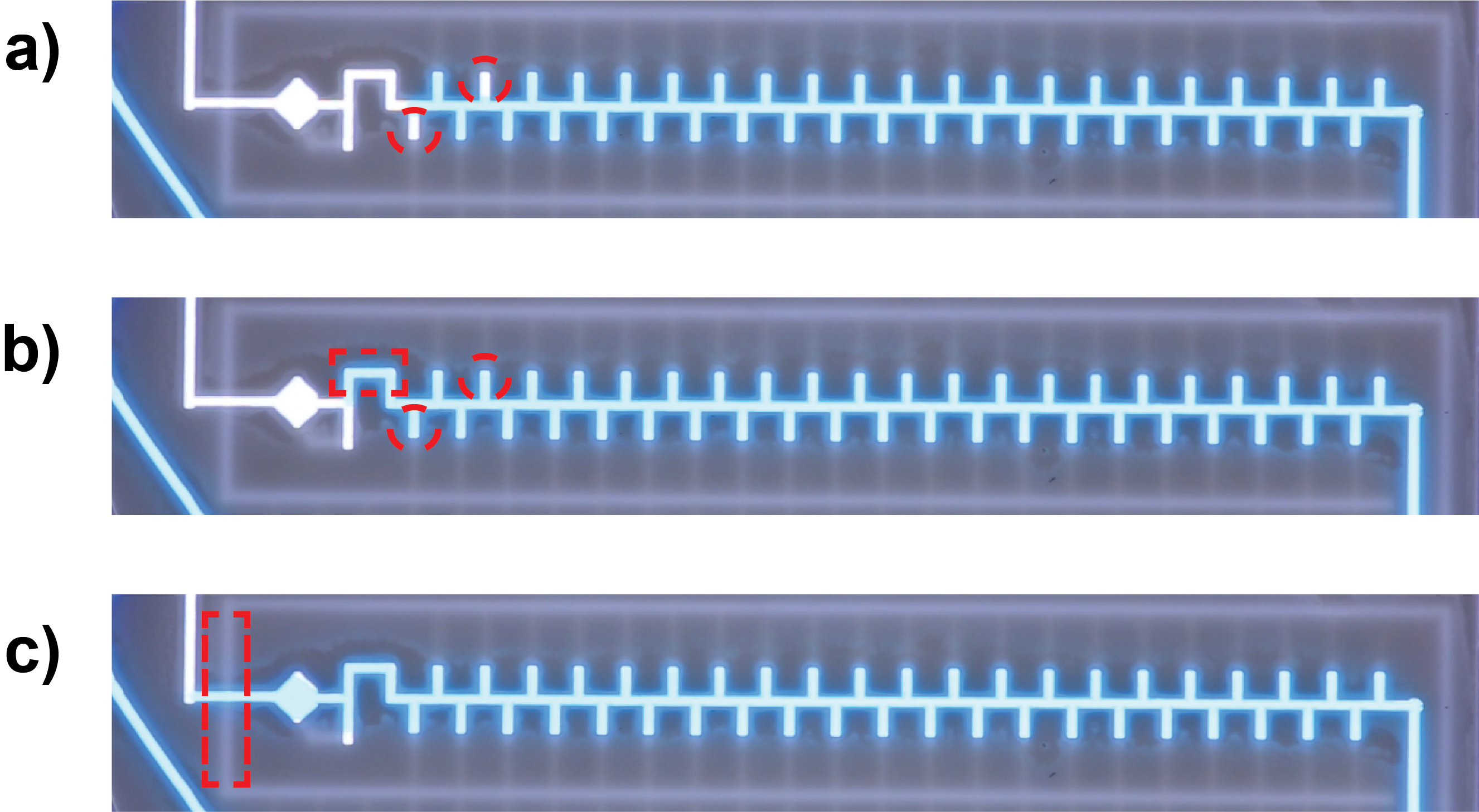
_**

**Figure S3.** Performance of the delay channel in ensuring complete filling of all MWs. **a** Some MWs take longer to fill with the sample solution due to their downstream position. **b** The delay channel allows sufficient time for all MWs to load successfully before sealing occurs. **c** The last branch of the primary vent (PV) is sealed with the sample solution only after all MWs are fully loaded.

**Note S4. Contact angle measurements of the water and different oils on different material layers of Cap-Drop**

Optimizing the performance of Cap-Drop requires a thorough understanding of fluid wettability on different substrate materials. To achieve this, contact angle measurements were performed for water, mineral oil, and mineral oil containing 1 CMC Span 20 surfactant on various adhesive materials and hydrophilic polyethylene terephthalate (PET) (**Figure S4**). The results, detailed in Table S1, offer critical insights into interfacial interactions, enabling the strategic selection of materials to enhance fluid manipulation within microchannels.


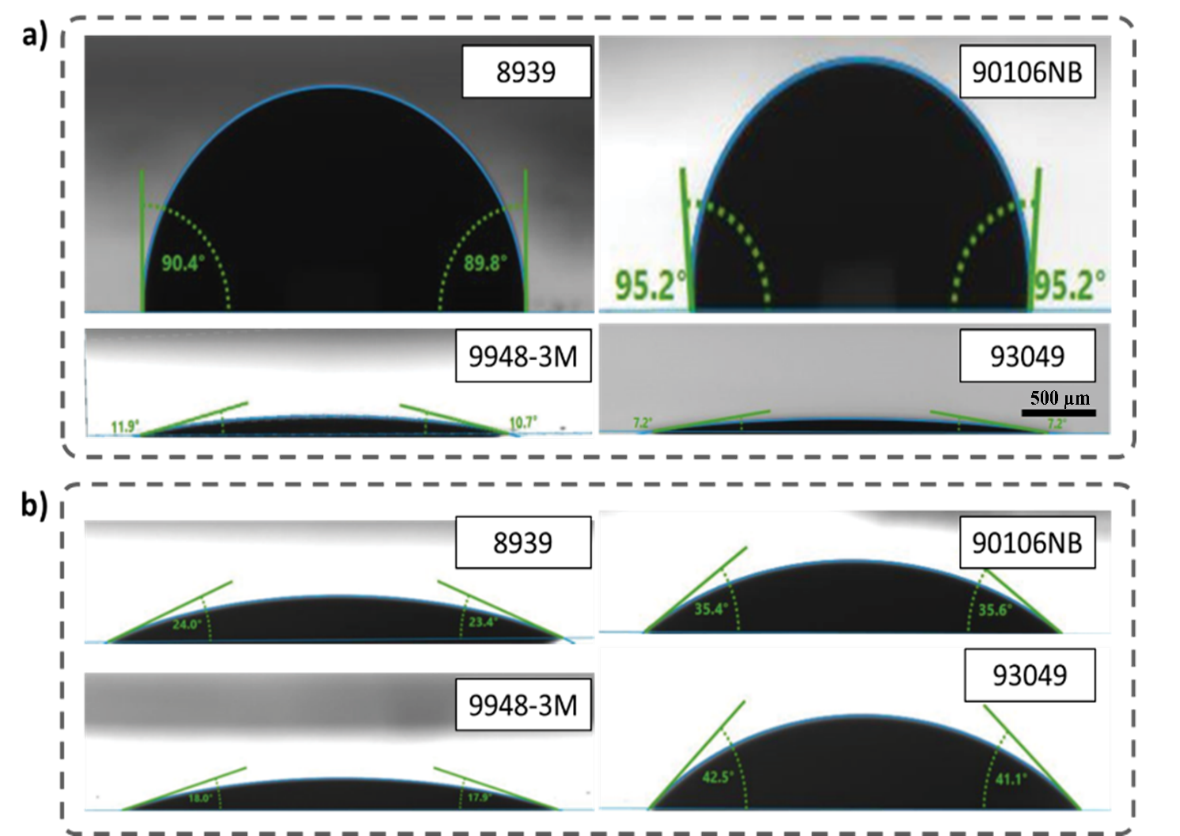


**Figure S4.** Static contact angle of AR care@8939, AR care@90106NB, 9984-3M, and AR care@93049 with **a** water and **b** mineral oil*. Scale bar: 500µm

**Table. S1.** Static contact angle of different adhesives and chip layers in contact with the water and different oils.

|  | **AR care@8939** | **AR care@90106NB** | **9984-3M** | **AR care@93049** |
| --- | --- | --- | --- | --- |
| **Contact angle- water** | 90.2º | 95.2º | 11.3º | 7.2º |
| **Contact angle-oil*** | 23.7º | 35.5º | 18º | 42º |
| **Transparency** | No | yes | yes | yes |
| **Explanation** | Double-sided ${PSA}^{\exists}$ | Double-sided ${PSA}^{\exists}$ | ${PET}^{\vartheta}$ | Single-sided ${PSA}^{\exists}$ |

$\exists$: Pressure-sensitive adhesive (PSA) $\vartheta:$Polyethylene terephthalate (PET) *: Mineral oil combined with one CMC span-20֒֒

# **Note S5. Effect of biofluid properties on fluid flow inside Cap-Drop**

**Table. S2.** Surface tension, viscosity, and static contact angle (θ_ca_) of various biofluids in contact with different materials used in chip layers.

|  | **Blood** | **Plasma** | **Saliva** | **Sweat** | **Water** |
| --- | --- | --- | --- | --- | --- |
| **θ_ca_ with AR-care@8939 (Adhesive)** | 98.9±1.03 º | 92.4±2.21 º | 101.9±0.28 º | 83.4±0.18 º | 91 º |
| **θ_ca_ with 9984 3M (Hydrophilic PET)** | 23.1±1.8 º | 20.2±0.7 | 15.5±1.0 º | 12.3±0.28 º | 8.8 ±1.24 º |
| **Surface tension (N/m)** | 54.38 ± 0.95 | 45.11 ± 0.23 | 60.89 ± 0.42 | 42.74 ± 0.61 | 71.88 ± 0.32 |
| **Viscosity (mp. s-1)** | 4.5 | 1.28 | 1.38 | 1 | 1 |

The flow behavior of biofluid within the MF channel is influenced by both the materials used for channel fabrication and the properties of the biofluid introduced at the inlet. **Figure S5** shows the biofluid interface within an MF channel with dimensions of 0.5 mm in width, 0.3 mm in height, and 20 cm in length. The channel's top and bottom layers are made of 3M 9984, while ARcare® 8939 is used for the adhesive layers forming the channel walls. Using the analytical method outlined in the optimization section, the biofluid interface progression was tracked over time. The results indicate that the velocity—represented by the slope of the interface curve—decreases as the channel fills with the sample solution. This decrease is attributed to the increasing channel resistance as the interface advances, while the capillary force remains constant.


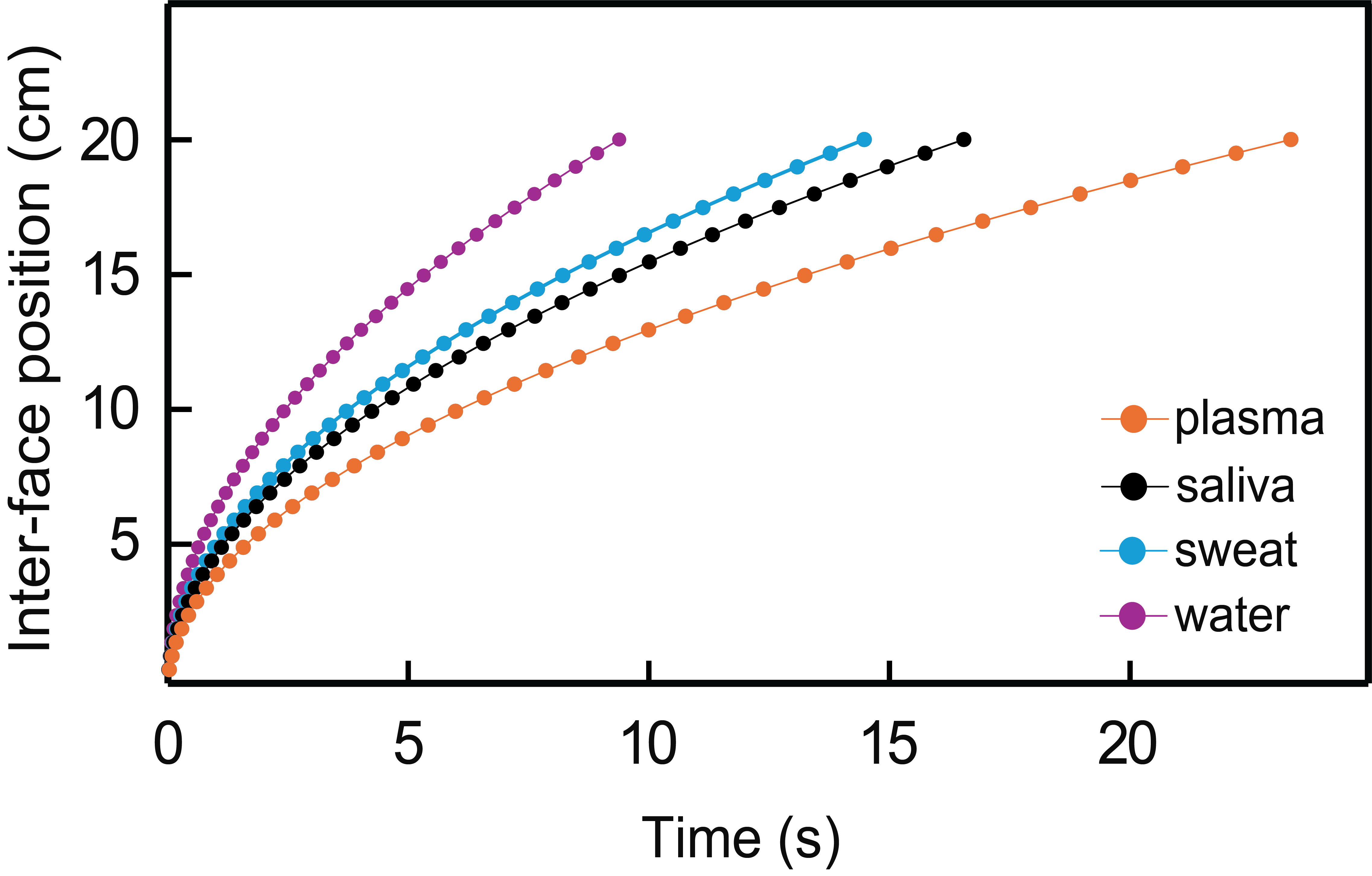


**Figure S5.** Interface position versus time for various biofluids (plasma, saliva, sweat, and water) in a microfluidic channel, used for measuring fluid velocity.

# **Note S6. Determining the stop valve’s and Wicking pad’s capillary pressure**

Capillary pressure (Δ𝑃) was calculated using the Young-Laplace equation (Eq. S1) to assess the performance of SV-3 in contact various biofluids used in this study, as illustrated in Figure 3.e.

$\Delta P=-\gamma\left[ \frac{\cos\theta_{t}+\cos\theta_{b}}{h}+\frac{\cos\theta_{l}+\cos\theta_{r}}{w} \right]$ *Eq. S1*

where w, h, γ, θt, θb, θr, and θl, respectively, represent the SV's width and height, liquid's surface tension, and top, bottom, right, and left-layer’s static contact angle.

To determine capillary pressure of Wp, we applied the Hagen-Poiseuille equation (Eq. S3). The hydraulic resistance for the rectangular channel was calculated using Eq. S2, and the flow rate was measured via image processing in Python (refer to Video S1).

$R=\frac{12\mu l}{wh^{3}\left[ 1-\frac{192h}{\pi^{5}w}+\sum_{n=1}^{\infty} \frac{1}{n^{5}}\tanh\frac{n\pi w}{2h} \right]}$ *Eq. S2*

$\Delta P=R.Q$ *Eq. S3*

The drag force acting on an air bubble moving through water in a microfluidic channel is determined using Stokes' drag equation (Eq. S4), applicable for low-velocity motion of small objects in a viscous fluid.

F_d_$=6\pi\mu rv$ *Eq. S4*

where Fd, $\mu$,r, and v denote drag force, dynamic viscosity of water, radius of the spherical air bubble, and velocity of the bubble relative to the water, respectively. The equation assumes laminar flow, a condition commonly met in microfluidic systems, and describes the viscous drag experienced by the bubble as it moves through the fluid.

**Note S7. Numerical method – COMSOL Multiphysics**

The functionality of Cap-Drop was simulated using the Level Set module in COMSOL, which offers significant advantages such as tracking interfaces with complex topologies and handling topological changes effortlessly. This module also integrates with other physics equations, making it highly versatile for modeling diverse problems. In this study, the capillary flow in the two phases is characterized by the Level Set variable (𝜙), which defines the position of the liquid-air interface and is described by Eq. S1. ^[1, 2]^

∂ϕ/∂t+u.∇ϕ=γ∇.(ε∇ϕ-ϕ(1-ϕ)n *Eq. S5*

n=∇ϕ/|∇ϕ|

where 𝑛 represents the normal to the air-water interface, 𝜖 denotes the interface thickness, and 𝛾 is the reinitialization parameter, set to the maximum velocity magnitude in the model. The variable 𝜙 ranges from 1 to 0, where 𝜙 > 0.5 (red) indicates regions occupied by water, and 𝜙 < 0.5 (blue) represents regions occupied by air. The flow rate 𝑢 corresponds to the laminar, incompressible fluid flow, governed by the unsteady continuity and momentum equations derived from the Navier-Stokes equations.

∇.u= 0 *Eq. S6*

ρ ∂u/∂t+ρ(u.∇)u= ∇.[-pI+μ(∇u+〖∇u〗^T )]+F_st

where ρ, μ, and p are density, dynamic viscosity, and flow pressure, respectively. The surface tension force F_st acting at the air-water interface is defined as Eq. S3.

F_st= ∇.T *Eq. S7*

T= σ(I-nn^T )δ

where 𝜎 represents liquid surface tension coefficient, and 𝛿 is the Dirac delta function, which is non-zero at the air-water interface. Additionally, the following equations are employed to calculate the density and viscosity of the fluids at the interface.

ρ= ρ_(ϕ=0) + (ρ_(ϕ=1) - ρ_(ϕ=0))ϕ *Eq. S8*

μ= μ_(ϕ=0) + (μ_(ϕ=1) - μ_(ϕ=0))ϕ

# **Note. S8 Cell distribution and coverage within MWs**


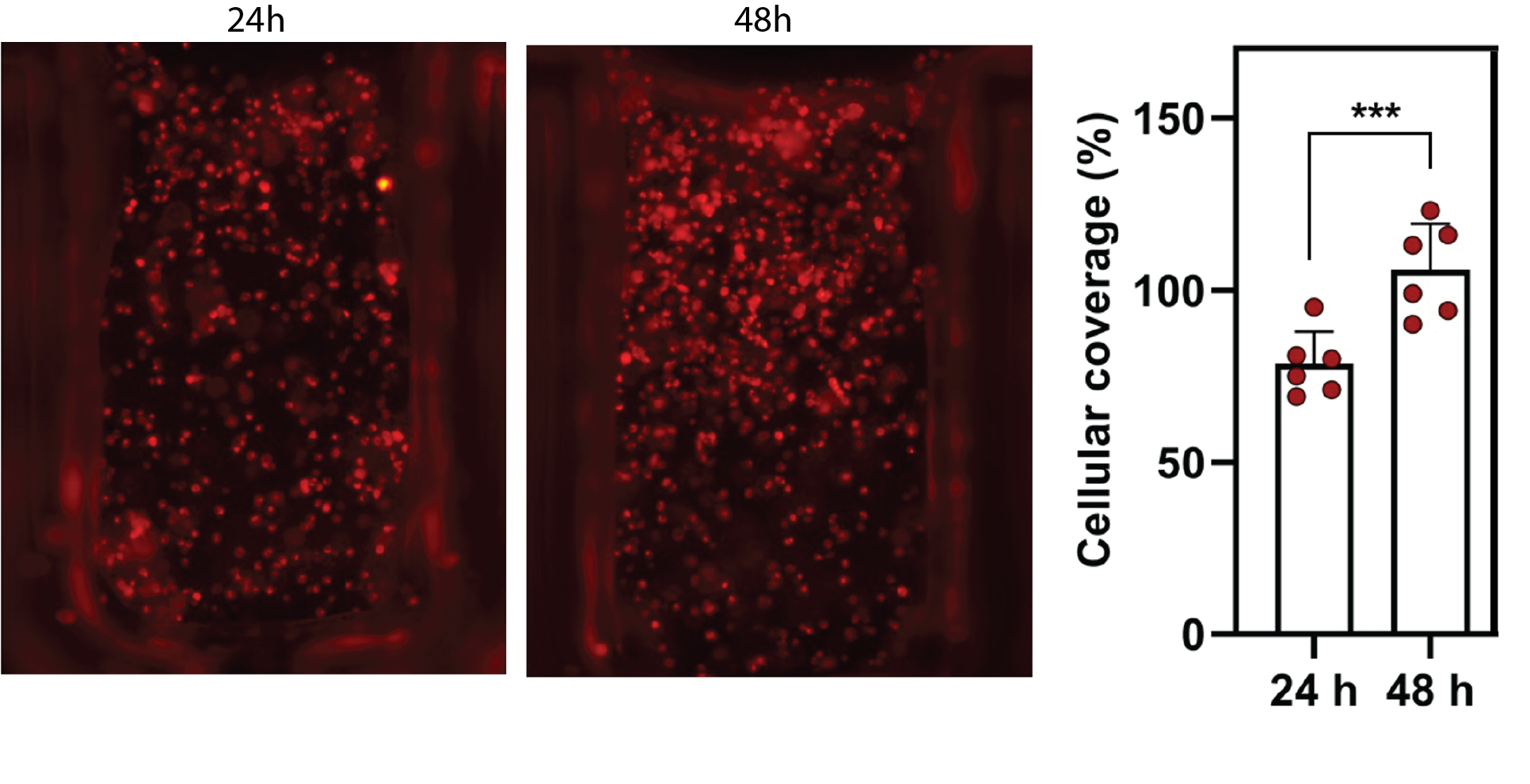


**Figure S6**. Uniform cell distribution and coverage within MWs, remains stable within 48 hours . Scale bare: 100 µm.

# **Note S9: Biocompatibility evaluation of hydrophilic PETs**


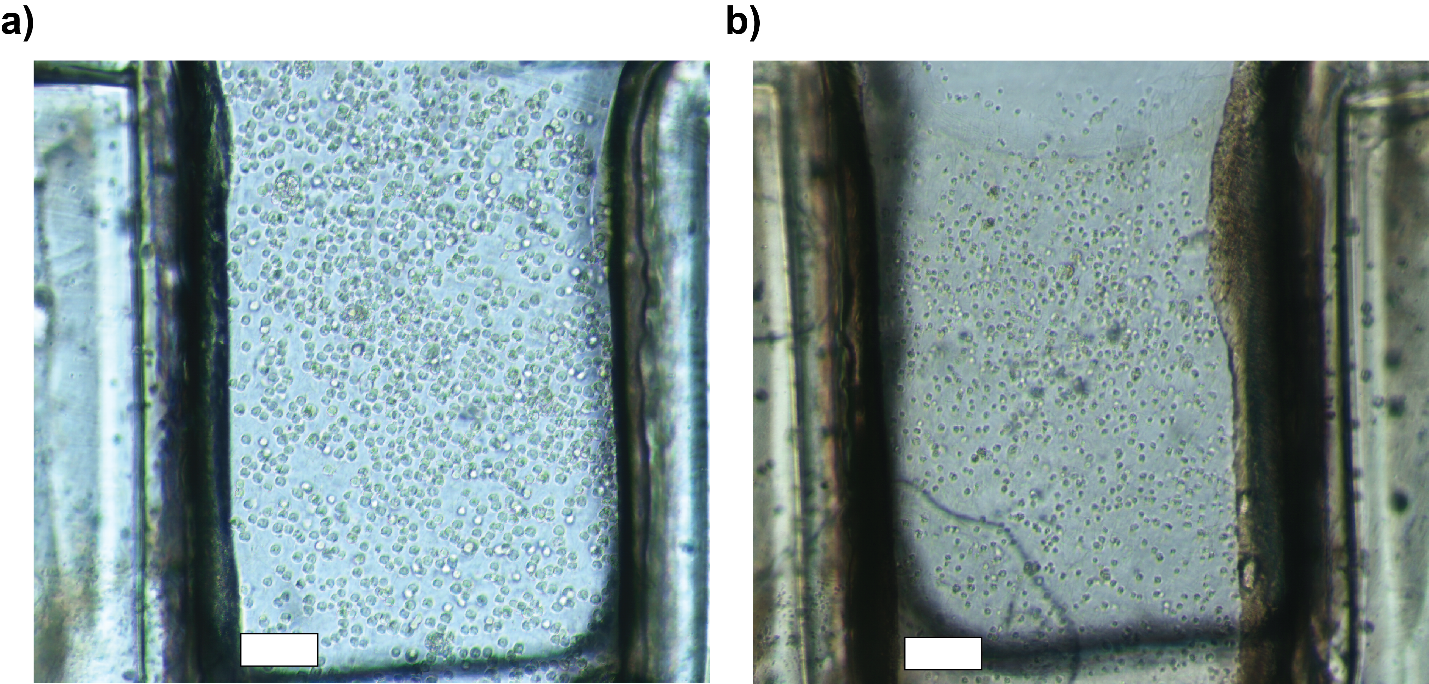


**Figure S7.** Biocompatibility evaluation of two hydrophilic PET materials for 3D cell culture**. a** 3M 9984 shows strong biocompatibility, with cells remaining healthy and viable after 24 hours. **b** ARcare® 93049 exhibits poor biocompatibility, as all cells appear shrunken and non-viable after 24 hours. Scale bar: 100 µm.

# **Note S10: Viscosity constraints and hydrogel compatibility in capillary-driven systems**

Among commonly used hydrogels, fibrillogenic crosslinking hydrogels such as collagen types I, III, and IV, ^[3, 4]^ fibrinogenic crosslinking hydrogels derived from fibrin-based precursors ^[5]^, thermally crosslinking hydrogels like Matrigel™, ^[6]^ photo-crosslinkable hydrogels (such as gelatin methacryloyl (GelMA) ^[7, 8]^), and alginate hydrogels ^[9]^ are generally compatible with capillary flow dynamics in Cap-Drop chips. However, the rheology of some hydrogels can pose challenges. Materials that do not exhibit shear-thinning behavior or possess inherently high viscosity increase the risk of clogging within the main channel. For example, highly crosslinked synthetic hydrogels like poly(2-hydroxyethyl methacrylate) (PHEMA) and polyacrylamides (PAMs) may absorb significant amounts of water, leading to swelling. ^[10, 11]^ This swelling induces compressive stresses that may cause the hydrogel layers to delaminate from their substrates, compromising the structural integrity of the device. Additionally, hydrogels such as unmodified hyaluronic acid (HA) and certain polyethylene glycol (PEG)-based formulations can exhibit high viscosities (>300 mPa·s) ^[12]^, which can hinder flow and uniform distribution. These effects are constrained by the principles of capillary flow as described by the Lucas–Washburn equation. ^[13]^

# **Note S11. Dimensional Parameters of the Cap-Drop-Cell Device**

**Table S3**. Dimensional parameters of the Cap-Drop-Cell

|  | **Parameter** | **Typical range** | **Selected value** |
| --- | --- | --- | --- |
| *1* | Main flow (MF) channel width | 0.4 – 0.7 mm | 0.6 mm |
| *2* | Microwell (MW) width | 0.4 – 0.8 mm | 0.5 mm |
| *3* | MW length – Adhesive layer 1 & 2 (L1) | L1≥ 0.5 mm (e.g., 0.5– 3 mm) | 1.0 mm |
| *4* | MW length – Adhesive layer 3 (L2) | L2≥ 0.5 mm (e.g., 0.5– 3 mm) | 1.2 mm |
| *5* | Pressure reducer length (L3 = L2 − L1) | L= L2 − L1 | 0.2 mm |
| *6* | MW and MF channel height | 0.2 – 0.4 mm | 0.3 mm |
| *7* | Stop valve (SV) width | 0.05 – 0.25 mm | 0.2 mm |
| *8* | Inlet diameter | – | 7 mm |
| *9* | Reservoir height | – | 5 mm |
| *10* | Distance between adjacent MWs (center-to-center) | – | 2.5 mm |
| *11* | Device dimension (width × length) | – | 35 mm × 70 mm |
| *12* | Device dimension (thickness) | – | 0.5 mm |

# **Note S12. Material stability and storage considerations for Cap-Drop**

The Cap-Drop device is fabricated from PET sheets and medical-grade pressure-sensitive adhesives (PSAs), such as 3M-9984 and ARcare® 90106NB. These materials are chemically stable, non-swelling, and impermeable to gases. According to manufacturer datasheets, the PSAs used in this device exhibit a shelf-life stability of 12–24 months under optimal storage conditions (21 °C, low humidity, and no direct sunlight). PET layers retain consistent surface energy, and PSA bonding integrity remains stable over time unless exposed to excessive heat or moisture.

In contrast to plasma-treated PDMS, which loses surface hydrophilicity over time, PET and PSA maintain stable surface properties, ensuring reliable capillary performance throughout their shelf life. As Cap-Drop is a single-use system, cumulative material fatigue is not a concern. To ensure consistent performance, the chip should be stored flat and sealed in a dry, room-temperature environment until use.

# **Note S13. Movies**

**Movie S1.** The capillary-driven workflow of Cap-Drop, including sample loading, fluid evacuation, and oil injection phases for sample digitization.

**Movie S2.** Demonstrating the operation of SVs, PV, and PRs across four consecutive microwells (MWs) in the Cap-Drop system.

**Movie S3.** The Cap-Drop-N workflow, featuring bubble-free loading achieved through normal vents (Norm-V) at the end of each MW. Also illustrates system failure during the evacuation phase, where air re-enters the chip via Wiki-Vs, particularly those closer to the WP.

**Movie S4.** Cap-Drop with MWs having a width of 0.2 mm causes slower flow movement compared to 0.5 mm widths, allowing us to observe the roughness effect and evaluate the effectiveness of the delay channel.

**Movie S5.** The bubble trap’s effectiveness in capturing air bubbles generated at the inlet.

**Movie S6.** The wicking pad’s absorption rate variations over time using OpenCV.

# **Note S14. Reporting summary**

## **Note S14.1 Data collection:**

The data was obtained using various instruments, including the Nikon Eclipse TE2000 optical microscope, the Nikon Eclipse Ti2 confocal laser scanning microscope, the Spectra MAX 190 plate reader, and a Canon EOS 6D camera.

## **Note S14.2 Data analysis**

Microsoft® Excel® for Microsoft 365 MSO (Version 2411, Build 16.0.18227.20002, 64-bit) was employed for graphing and statistical analysis. Python 3.12.2 with the OpenCV library (version 4.9.0) was employed for image processing. MATLAB R2021b was utilized for coding and analytical simulations, while COMSOL Multiphysics 6.1 was used for numerical simulations.

## **Note S14.3 Sample size and replication**

(1) Fig. 2d: The absorbance power of the wicking pad was analyzed using Python code based on observations from three independent experiments, yielding n = 3 independent replicates. As no external variable affects its power, three replicates were deemed sufficient.(2) Fig. 3c: The sample size included 18 different widths, ranging from 0.2 mm to 2.5 mm, with n = 4 replicates for each width. The experiment was repeated for two different biofluids: one representing high-viscosity flow (citrated whole blood) and the other representing low-viscosity flow (water).(3) Fig. 3d: The sample size included three groups of microwells with varying average volumes: 100 microwells with an average volume of 300 nL, 100 microwells with an average volume of 175 nL, and 30 microwells with an average volume of 40 nL. Each group was tested in n = 3 independent experiments to assess volume variance.(4) Fig. 4a,b: The sample size included three independent experiments (n = 3), each with 100 replicated microwells filled with three different concentrations of citrated whole blood. Results were analyzed using Python.(5) Fig. 4c: The sample size included two independent experiments (n = 2), each with 100 replicated microwells filled with two different particles of sizes 15 µm and 10 µm. (6) Fig. 4d: The sample size included 100 replicated microwells filled with Caco-2 cells, yielding n = 100 replicates. (7) Fig. 5e: The sample size consisted of two independent experiments (n = 2), with 100 microwells replicated in each. For data collection, 12 microwells were chosen at random from each experiment. (8) Fig. 5d: Two independent experiments (n = 2) were conducted, each comprising 100 replicated microwells. From each experiment, 12 microwells were randomly selected for analysis.

## **Note S14.4 Randomization**

For all figures, we analyzed all frames and microwells. However, for Figures 5d and 5e, 12microwells were randomly selected for analysis of live/dead cell viability and cell proliferation. The selection was performed using Python's random sample function, which randomly chose five microwells from a pool of 100 microwells, ensuring an unbiased and reproducible process.

## **Note S14.5 Blinding**

•Fig. 4a and 4d: Four chips, all with the same dimensions and an average volume of 300 nL, were used—three for blood testing and one for determining cell numbers across microwells. Two chips were fabricated by the first author, while the third and fourth chips were prepared by the third and fifth authors, respectively.

•Fig. 5e: To assess cell viability across 100 microwells, two independent experiments were conducted. One experiment was performed by the second author, and the other by the sixth author.

•Fig. 5d: To evaluate cell proliferation among 100 microwells, two independent experiments were carried out. One was conducted by the second author, and the other by the sixth author.

## **Note S14.6 Data exclusion**

No exclusion,

## **Note S14.7 Antibodies**

1. MKI67 monoclonal antibody, Sigma-Aldrich, WH0004288M1-100UG, L9161-7B8

<https://www.sigmaaldrich.com/CA/en/product/sigma/wh0004288m1>

1. Goat anti-mouse IgG2a (H+L) Aexa Fuor™ 647, Invitrogen ThemoFisher Scientific, A21241, 2482961

<https://www.thermofisher.com/antibody/product/Goat-anti-Mouse-IgG2a-Cross-Adsorbed-Secondary-Antibody-Polyclonal/A-21241>

1. Antibodies in the Human Cortisol Competitive ELISA Kit

<https://www.thermofisher.com/elisa/product/EIAHCOR.html?gclid=Cj0KCQiAw8OeBhCeARIsAGxWtUzy0XjMXDaQJtoqyu5RHbGRGmNinuxnfDv2ZojxOdREZBVolJSn3coaAoxeEALw_wcB&ef_id=Cj0KCQiAw8OeBhCeARIsAGxWtUzy0XjMXDaQJtoqyu5RHbGRGmNinuxnfDv2ZojxOdREZBVolJSn3coaAoxeEALw_wcB:G:s&s_kwcid=AL!3652!3!605739441808!e!!g!!cortisol%20elisa%20kit!352133802!40402348982&cid=bid_pca_iel_r01_co_cp1359_pjt0000_bid00000_0se_gaw_nt_pur_con>

## **Note S14.8 cell line source**

1. Caco-2 (ATCC HTB-37™) No primary culture, and no participant.

Authenticated by ATCC and Sigma-Alddrich. Please see the links below;

<https://www.atcc.org/products/htb-37>

## **Note S14.9 Ethics oversight**

#REB16-1147, issued by the University of Calgary Research Ethics Board.

Male, 31 year old.

**References**

[1] G.-H. Cottet, E. Maitre, T. Milcent, *Level Set Methods for Fluid-Structure Interaction, Vol. 210*, Springer Nature, **2022**.

[2] S. Mehraji, M. Saadatmand, *Physics of Fluids* **2021**, *33*, 072009.

[3] S. Maji, H. Lee, *International Journal of Molecular Sciences* **2022**, *23*, 2662.

[4] A. Vedadghavami, F. Minooei, M. H. Mohammadi, S. Khetani, A. R. Kolahchi, S. Mashayekhan, A. Sanati-Nezhad, *Acta biomaterialia* **2017**, *62*, 42-63.

[5] H. A. Elshabrawy, H. A. Moustafa, M. H. Yacoub, I. M. El-Sherbiny, *Emergent Materials* **2024**, 1-24.

[6] A. Passaniti, H. K. Kleinman, G. R. Martin, *Journal of cell communication and signaling* **2022**, *16*, 621-626.

[7] S. Das, J. T. Jegadeesan, B. Basu, *Biomacromolecules* **2024**, *25*, 2156-2221.

[8] K. Yue, G. Trujillo-de Santiago, M. M. Alvarez, A. Tamayol, N. Annabi, A. Khademhosseini, *Biomaterials* **2015**, *73*, 254-271.

[9] Y. Zhang, C. An, Y. Zhang, H. Zhang, A. F. Mohammad, Q. Li, W. Liu, F. Shao, J. Sui, C. Ren, *Materials Science and Engineering: C* **2021**, *131*, 112497.

[10] S. S. Velankar, V. Lai, R. A. Vaia, *ACS applied materials & interfaces* **2012**, *4*, 24-29.

[11] N. Annabi, J. W. Nichol, X. Zhong, C. Ji, S. Koshy, A. Khademhosseini, F. Dehghani, *Tissue Engineering Part B: Reviews* **2010**, *16*, 371-383.

[12] H. V. Le, D. Le Cerf, *Small* **2022**, *18*, 2204283.

[13] J. Cai, T. Jin, J. Kou, S. Zou, J. Xiao, Q. Meng, *Langmuir* **2021**, *37*, 1623-1636.
